# Supplementary material for: Reprogramming Müller glia to regenerate ganglion-like cells in adult mouse retina with developmental transcription factors
Source: Sci Adv. 2022 Nov 23;8(47):eabq7219. doi: 10.1126/sciadv.abq7219 (PMC9683702; doi:10.1126/sciadv.abq7219)
Supplement: 20221123-1 [file sciadv.abq7219.v1.pdf]

DEVELOPMENTAL BIOLOGY

Reprogramming Müller glia to regenerate ganglion-like cells in adult mouse retina with developmental transcription factors

Copyright © 2022 The Authors, some rights reserved; exclusive licensee American Association for the Advancement of Science. No claim to original U.S. Government Works. Distributed under a Creative Commons Attribution License 4.0 (CC BY).

Levi Todd[1]†, Wesley Jenkins[1]†, Connor Finkbeiner[1], Marcus J. Hooper[1], Phoebe C. Donaldson[1], Marina Pavlou[1], Juliette Wohlschlegel[1], Norianne Ingram[2], Fred Rieke[2], Thomas A. Reh[1]*

Many neurodegenerative diseases cause degeneration of specific types of neurons. For example, glaucoma leads to death of retinal ganglion cells, leaving other neurons intact. Neurons are not regenerated in the adult mammalian central nervous system. However, in nonmammalian vertebrates, glial cells spontaneously reprogram into neural progenitors and replace neurons after injury. We have recently developed strategies to stimulate regeneration of functional neurons in the adult mouse retina by overexpressing the proneural factor Ascl1 in Müller glia. Here, we test additional transcription factors (TFs) for their ability to direct regeneration to particular types of retinal neurons. We engineered mice to express different combinations of TFs in Müller glia, including Ascl1, Pou4f2, Islet1, and Atoh1. Using immunohistochemistry, single-cell RNA sequencing, single-cell assay for transposase-accessible chromatin sequencing, and electrophysiology, we find that retinal ganglion–like cells can be regenerated in the damaged adult mouse retina in vivo with targeted overexpression of developmental retinal ganglion cell TFs.

INTRODUCTION

Neurodegenerative disorders of the eye result in blindness because the mammalian nervous system lacks a regenerative capacity. In other vertebrates, such as fish and amphibians, the retina is able to replace lost neurons and restore visual function (*1*). Müller glia (MG), the primary glial cell in the vertebrate retina, can serve as a source of neurogenic progenitors in regenerative species (*2*). However, in the mammalian retina, MG respond to retinal damage by undergoing an inflammatory response instead of a regenerative one (*3*).

In the past few years, our group and others have found strategies to stimulate MG in adult mice to behave similar to their fish counterparts and generate neurons (*4*–*9*). We screened a number of transcription factors (TFs) that were differentially expressed between mouse MG and retinal progenitors for their ability to stimulate neurogenesis in MG in vitro (*10*). One factor that emerged from this screen was the proneural basic helix-loop-helix (bHLH) TF Ascl1, which was able to induce neurogenesis from MG in vivo in young mice (*11*) and when cotreated with a histone deacetylase [trichostatin A (TSA)] in adult mice (*4*). Ascl1-expressing MG adopt a molecular phenotype similar to developing retinal progenitors, and a subset of these cells undergoes mitotic division (*5*, *12*). Some of these newly generated cells go on to differentiate into retinal neurons that connect with the endogenous circuitry (*4*, *5*). Using Ascl1 to stimulate MG neurogenesis causes most MG-derived neurons to take on a bipolar cell fate, with a minority resembling amacrine cells (*4*, *12*). Recently, we reported that the efficiency and range of neuronal cell types generated through MG reprogramming can be substantially improved by adding an additional bHLH TF of the atonal class (Atoh1/7). With this combination, up to 80% of the MG expressing Ascl1 and Atoh1 will become neurogenic precursors and ultimately neurons (*13*).

Our previous results show that combining two TFs of the same type (bHLH proneural) causes a substantial increase in the efficiency of in vivo MG reprogramming, but we were unable to control the types of neurons generated by MG in this paradigm. This is in some way similar to the process of regeneration in zebrafish, where injury triggers the generation of all neuronal types, regardless of whether the injury is widespread or targeted to specific cell types (*14*, *15*). However, particular retinal diseases often are the result of a defect in a specific neural class. For example, in glaucoma, blindness results from the loss of retinal ganglion cells (RGCs) (*16*). Therefore, an impetus exists to direct endogenous regeneration to a particular affected type of neuron for cell replacement strategies.

Here, we test whether combining Ascl1 with other types of developmentally important TFs can more precisely direct MG-derived retinal progenitors to specific retinal cell fates. Specifically, we explore the effects of combining Ascl1 overexpression with two other TFs of different classes, Islet1, a LIN, Islet1, MEC3 (LIM) homeodomain TF, and Pou4f2, a class IV, Pit-Oct-Unc (POU) homeodomain TF. Both factors have been shown to be important for cell fate determination in the developing retina, with Pou4f2 necessary for RGC differentiation and Islet1 important in the development of several types of retinal neurons including RGCs (*17*, *18*).

We find that the expression of Pou4f2 and Islet1, along with Ascl1, in adult mouse MG, directs a subset of the neurons generated by the MG toward a cell fate that resembles RGCs. The MG-derived RGC-like neurons (i) can be immunolabeled with markers of normal RGCs; (ii) have a transcriptome similar to developing RGCs by single-cell RNA sequencing (scRNA-seq); (iii) have a broader range of electrophysiological characteristics than neurons generated by Ascl1 alone, such as action potentials; and (iv) display a pattern of chromatin accessibility similar to developing

[1]Department of Biological Structure, University of Washington, Seattle, WA 98195, USA. [2]Department of Physiology and Biophysics, University of Washington, Seattle, WA 91895, USA.
*Corresponding author. Email: tomreh@uw.edu
†These authors contributed equally to this work as co–lead authors.

RGCs. Together, our results show that neural regeneration from MG can be directed to specific cell types using combinations of developmentally relevant TFs.

RESULTS

Pou4f2 and Islet1 increase the diversity of neurons from Ascl1-reprogrammed MG

We previously developed a mouse where Ascl1 is induced specifically in MG (*Glast-CreER:LNL-tTA:teto-mAscl1-GFP:ccGFP*) by application of tamoxifen. After Ascl1 induction, retinal injury [*N*-methyl-D-aspartate (NMDA)] followed by injection of a histone deacetylase inhibitor (TSA) induces MG to express genes associated with developing retinal progenitors and to generate new neurons in the adult mouse retina in vivo. Most of these newly generated neurons adopt a bipolar cell fate (*4*, *5*, *12*). To examine whether expression of additional TFs (Pou4f2 or Islet1) can direct the MG-derived progenitors to other neuronal fates, we crossed the *Glast-CreER:LNL-tTA:teto-mAscl1-GFP* mice with a *tetO-Pou4f2-tetO-Islet1* transgenic mouse line (tetO-IPA; Fig. 1A). This construct encodes *Pou4f2* and *Islet1* separated by two different loxP variants (*19*). When exposed to Cre recombinase, this cassette allows for expression of either *Pou4f2*, *Islet1*, or sometimes both.

The tetO-IPA mouse line allows us to test whether MG reprogramming is enhanced by treatment with Pou4f2 + Ascl1, Islet1 + Ascl1, or Islet1 + Pou4f2 + Ascl1 (hereafter IPA). After intraperitoneal application of tamoxifen to induce the TFs in MG, we induced a retinal injury by intravitreal injection of NMDA, followed by TSA (Fig. 1B). The mice were then euthanized 3 weeks later for immunofluorescence analysis to assess the fate of recombined MG (Fig. 1, C to E). Figure 1 (C and D) shows that this protocol induces the transgenes in MG and neurons derived from them [green fluorescent protein positive (GFP^+)]. We found GFP^+ MG-derived cells expressing either Brn3 (Pou4f2) (17%), Islet1 (33%), or both TFs (7%) (Fig. 1, C and D).

Additional immunohistochemistry (IHC) analysis demonstrated that the IPA combination effectively promoted neurogenesis from MG. Most glial-derived cells acquire a neuronal morphology 3 weeks after injury (Fig. 1E). Quantifications of MG-derived cells in retinal sections from IPA-treated mice confirmed that the MG-derived GFP^+ neuronal-like cells expressed the ganglion/amacrine marker HuC/D (Fig. 1, F and G) or the bipolar marker Otx2 (Fig. 1, G and H). IPA expression substantially enhanced MG neurogenesis of HuC/D neurons compared to Ascl1 alone (Fig. 1G). Consistent with our previous reports, a subset of the MG-derived neurons was derived from EdU^+, proliferating MG (fig. S1, A to C). Together, these data suggest that the addition of TFs Pou4f2 and/or Islet1 enhances the neurogenic capacity and expands the resulting cell fates of Ascl1-MG in vivo.

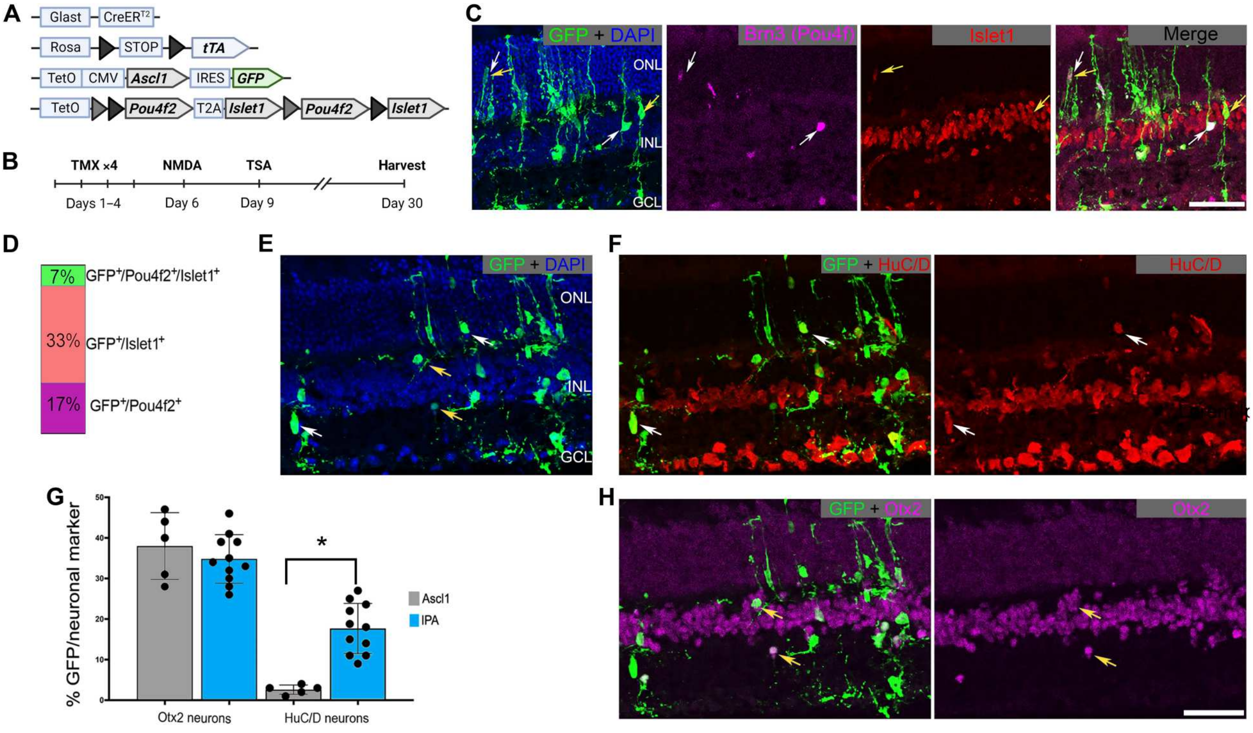

Fig. 1. Pou4f2 and/or Islet1 stimulate regeneration of RGC-like neurons. (**A**) Schematic depicting the transgenic constructs used to induce Ascl1 and Pou4f2/Islet1 specifically in MG. Pou4f2/Islet1 is surrounded by mutually exclusive floxed sites, leading to expression of Pou4f2, Islet1, or both in the presence of active Cre. (**B**) Experimental paradigm to induce retinal regeneration in adult mice. Tamoxifen (TMX). (**C**) Representative sections of the retina after intravitreal NMDA damage, showing transgenic expression of Pou4f2/Brn3 (purple) and/or Islet1 (red) in GFP^+ lineage–traced MG. DAPI, 4′,6-diamidino-2-phenylindole. (**D**) Quantification of the percent of transgene-expressing MG that express Pou4f2, Islet1, or both. (**E** and **F**) Representative sections showing MG-derived neurons after the regeneration paradigm expressing HuC/D (red). (**G**) Quantification of the percent of GFP^+ MG-derived neurons that express either HuC/D or Otx2. (**H**) Examples of MG-derived neurons expressing Otx2 (purple). Significance of difference was determined using an unpaired *t* test (asterisk = $p < 0.0001$); dots represent individual animals. Scale bars, 50 μm. ONL, outer nuclear layer; INL, inner nuclear layer; GCL, ganglion cell layer. Mouse schematic was made with Biorender.com.

We had previously found that the combination of Ascl1:Atoh1 could stimulate neurogenesis from MG in the absence of retinal damage (*13*). In contrast, IPA induction in the undamaged retina was not sufficient to stimulate neurogenesis (fig. S2, A, B, and E). We also tested whether MG could undergo neurogenic reprogramming if we activated the IPA factors in MG after NMDA injury and TSA rather than before NMDA and TSA as shown in Fig. 1. We found that induction of IPA after injury stimulated a small amount of neurogenesis, but substantially less than our original paradigm (fig. S2, C to E).

Because a substantial portion of the newly generated neurons in the damaged retina expressed the ganglion/amacrine marker HuC/D, we performed whole-mount imaging of IPA-derived neurons to better assess their neuronal morphology. This revealed cells with large branching dendritic arbors reminiscent of RGCs or wide-field amacrine cells; cells with this morphology were not previously seen with Ascl1 alone (Fig. 2, A to F).

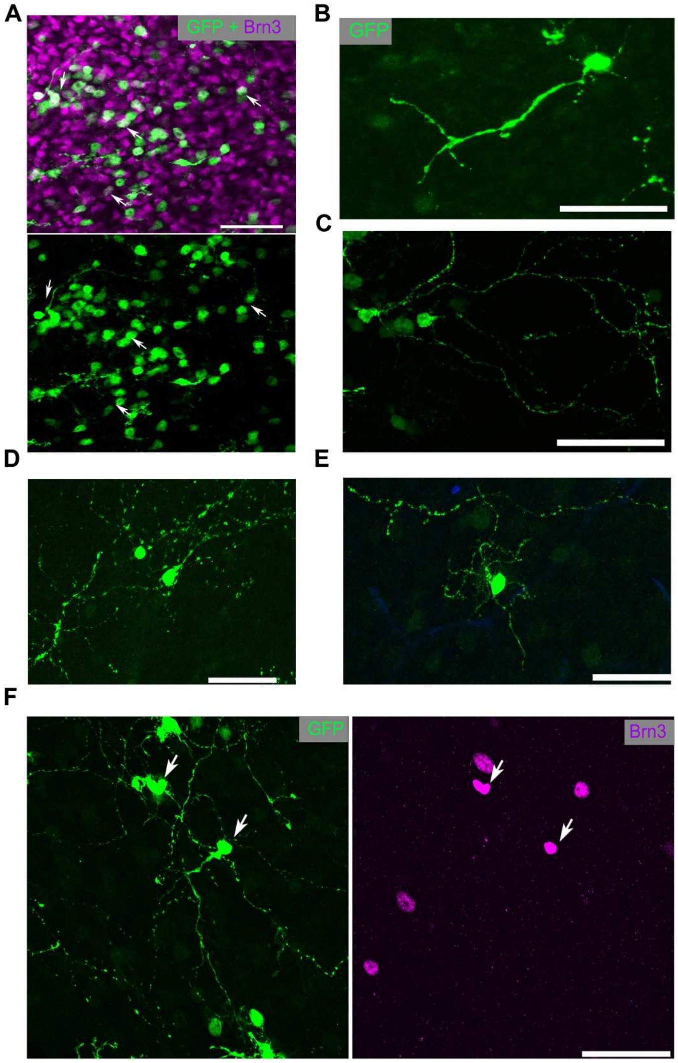

Fig. 2. IPA-stimulated MG-derived neurons display complex neuronal morphology. (**A**) Retinal whole mounts stained for GFP (MG-derived cells; green) and Brn3 (purple). (**B** to **E**) Examples of the morphology of GFP$^+$ MG-derived cells. (**F**) MG-derived (GFP$^+$) cell with complex neurites colabeled with Brn3 (purple). Scale bars, 50 μm.

IPA induces neurons with an RGC-like transcriptome

We next used scRNA-seq to analyze how Islet1 and Pou4f2 alter the phenotype of Ascl1-mediated MG reprogramming. Three weeks after initiating the IPA regeneration protocol, MG cells and their progeny were fluorescence-activated cell sorting (FACS)–purified and processed for scRNA-seq as previously described (*5*, *12*). To directly compare the changes in cell fates induced by IPA with those caused by expression of Ascl1 alone, we used Seurat to integrate data from IPA treatment with previously obtained Ascl1-only reprogramming libraries (*4*) and clustered the cells (Fig. 3A). The combined data from the IPA experiment and the prior Ascl1 dataset were projected onto a single uniform manifold approximation and projection (UMAP) plot and clusters of cell types were identified by known marker genes (Fig. 3B).

The combined UMAP plot of Ascl1-only versus IPA treatment contains clusters of cell types (e.g., MG, progenitors, and bipolar cells) that we have previously observed during Ascl1-mediated reprogramming (Fig. 3A). This analysis revealed two additional phenotypes unique to the IPA condition. First, the neurogenic efficiency of MG is increased over twofold with IPA versus Ascl1 only (Fig. 3C), which is consistent with our IHC data and suggests that the combination of these three TFs potently stimulates neurogenesis from adult MG. Second, a novel cluster appeared after IPA treatment that did not exist in the Ascl1-only condition (green cluster) (Fig. 3C). While cells expressing Islet1 but not Pou4f2 were found mostly in the bipolar cluster, cells expressing Pou4f2, with or without detectable Islet1, were largely found in the novel RGC-like cluster (fig. S3, A to C).

The new cluster of cells induced by IPA shows a high expression of genes characteristic of RGCs, such as *Elavl4* and *Sox11* (Fig. 3B) (*20*). In addition, we found that these IPA neurons expressed many genes found in the gene ontology (GO) terms "axon guidance," "axon outgrowth," and "axogenesis" (fig. S3D). Because Islet1 and Pou4f2 are upstream of an RGC fate–inducing regulatory network, we assayed whether this combination of factors was able to induce multiple RGC genes in this cluster of MG-derived neurons. We used the label transfer feature of Seurat to broadly compare the transcriptome of the IPA neurons to a reference dataset of all major retinal neuron classes (*21*, *22*). The novel cluster was classified as an RGC cluster (average prediction score = 0.74), suggesting an overall transcriptomic similarity to native RGCs (fig. S3E). Compared to Ascl1-induced neurons, a substantial number of RGC-associated genes were expressed in IPA-induced neurons (Fig. 3D). These include genes such as *Sox4* and *Sox11*, which are redundantly required for RGC fate acquisition (*23*, *24*), and the axon growth–associated gene *Gap43*, which is highly expressed in developing RGCs (*25*). We found that both *Satb1* and *Cntn5* were expressed in subsets of IPA-induced neurons. *Satb1* is highly expressed in the ON-OFF direction-selective subtype of RGCs where it controls *Cntn5* expression (*26*). Using IHC, we were able to confirm that IPA-induced neurons expressed Satb1 protein (Fig. 3E). In addition, the RGC and amacrine marker Calretinin (*Calb2*) was also detected at the RNA and protein levels (Fig. 3, C and F).

Despite this large suite of RGC genes expressed by the MG-derived RGC-like neurons, these cells fail to express some canonical RGC markers such as *Pou4f1*, *Sncg*, *Rbpms*, and *Nefm* (Fig. 3D).

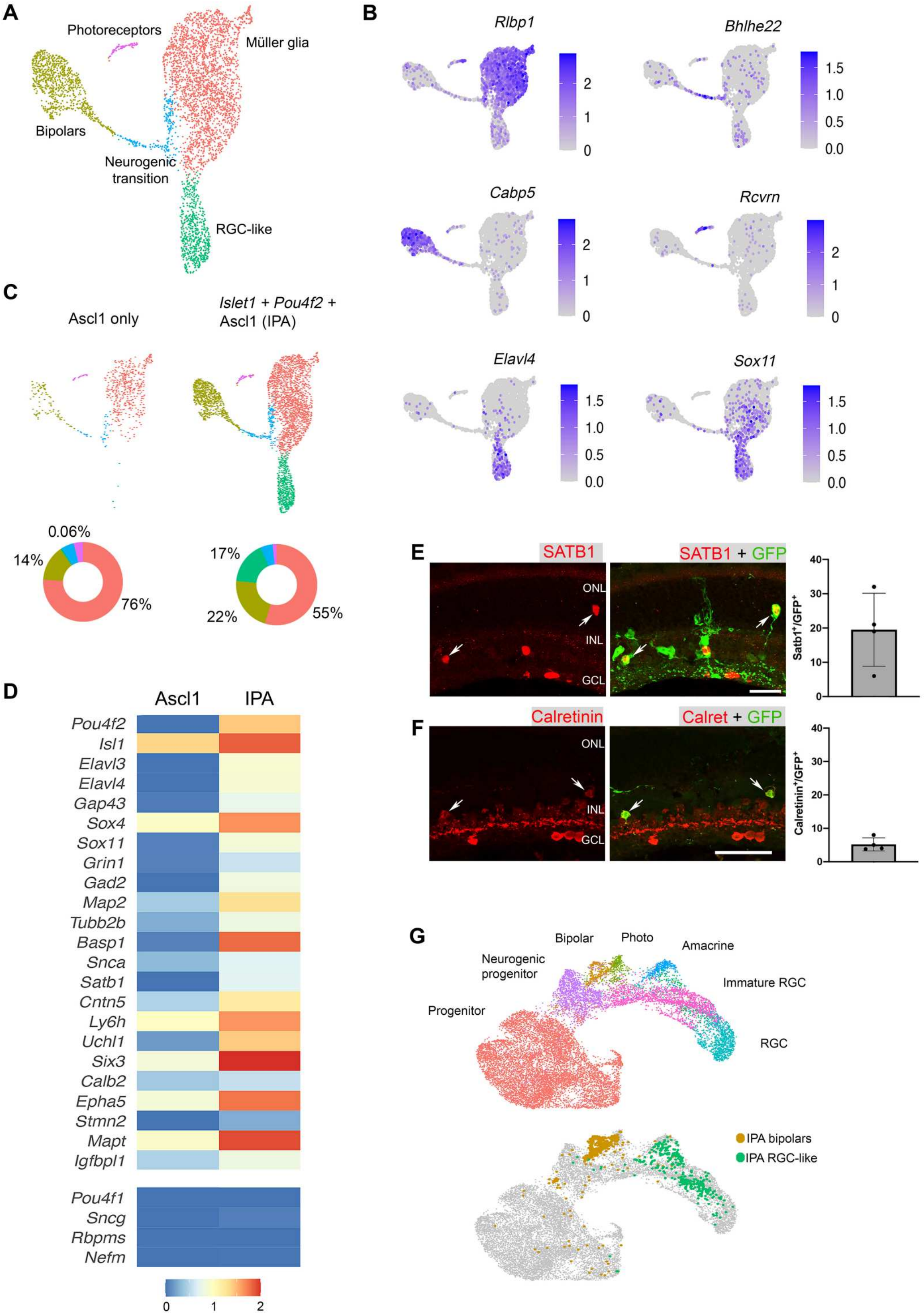

Fig. 3. scRNA-seq analysis of Pou4f2/Islet1-stimulated neurons reveals molecular characteristics of RGCs. (**A**) UMAP plot for FACS-sorted MG-derived cells after the IPA regeneration paradigm combined with a previous scRNA-seq dataset where Ascl1 only was used. (**B**) Feature plots highlight the major clusters of MG (*Rlbp1*), neurogenic transition (*Bhlhe22*), bipolars (*Cabp5*), photoreceptors (*Rcvrn*), and RGC-like cells (*Elavl4* and *Sox11*). (**C**) The distribution of cells from each treatment projected onto a split UMAP plot. Donut plots represent the percent each cluster comprises of the dataset. (**D**) Heatmap comparing scRNA-seq datasets of Ascl1 only versus IPA treatment. Selected genes are depicted that are associated with RGCs. (**E**) Retinal sections stained for MG-derived cells (GFP) with Satb1 (red), and quantifications show the percent of GFP$^+$ cells that are Satb1$^+$. (**F**) Retinal sections stained for MG-derived cells (GFP) with Calretinin (red), and quantifications show the percent of GFP$^+$ cells that are Calretinin$^+$. (**G**) UMAP of IPA-derived neurons integrated with scRNA-seq of E14 mouse retina from Clark *et al.* (*21*), revealing that IPA neurons cluster similarly to immature RGCs. Scale bars, 50 μm.

This suggests that the newborn neurons do not fully differentiate into mature RGCs. Consistent with this observation, when we integrated the scRNA-seq data of IPA-induced neurons with a dataset from E14 (embryonic day 14) embryonic mouse retina (*21*), we find that the MG-derived RGC-like neurons most closely resemble immature RGCs (Fig. 3G). We compared the IPA-induced neurons from a 3-week end point with a longer survival time point (6 weeks) and found that the regenerated RGC-like neurons were a stable population and did not show evidence of increased cell death or stress over this period (fig. S4, A to D).

Simultaneous expression of Islet1-Pou4f2 with Ascl1 more uniformly induces an RGC state from MG in vitro

Because the tetO-Pou4f2 and Islet1 transgenic construct contains mutually exclusive loxP sites, most of the Cre-expressing MG in vivo expressed Ascl1 and either Pou4f2 or Islet1. While a small number of cells colabeled for both Pou4f2 and Islet1 in vivo (Fig. 1, C and D), to better assess the effect of overexpressing all three transgenes uniformly, we bred mice containing the tetO-Pou4f2-Islet1 and tetO-Ascl1-GFP cassettes to a germline Rosa26-rtTA line and then performed in vitro MG-reprogramming experiments (Fig. 4, A and B). MG were cultured from postnatal day 1111 mice for 7 days before passaging as previously described (*10*). The cultures obtained in this way are largely composed of MG, although some surviving neurons are observed (*10*). 5-Ethynyl-2′-deoxyuridine (EdU) was added to the medium to determine which cells are derived from proliferating MG and which cells were likely surviving neurons from the initial dissociation. After passage, doxycycline was added to the medium to induce transgene expression, and then cells were assayed with immunofluorescence and scRNA-seq (Fig. 4B).

Analysis of the cultures after 5 days of treatment confirmed that the cells express the transgenes. Immunolabeling for the transgenes showed that cells coexpress Pou4f2, Islet1, and Ascl1 (fig. S5A). The cells largely adopted a neuronal morphology, and immunolabeling demonstrated EdU^+ cells that were colabeled with neuronal markers Tuj1 (Fig. 4C), Neurofilament (Fig. 4D), and Calbindin (Fig. 4E).

To determine how the overexpression of IPA differed from Ascl1 alone, we repeated the reprogramming experiment as described above, alongside sister MG cultures from tetO-Ascl1;Rosa26-rtTA mice, either with or without doxycycline treatment to activate transgene expression. We carried out scRNA-seq as described above for each sample. The untreated MG were largely homogeneous, with one glial cluster and a small cluster containing only a few surviving neurons (fig. S5, B and C). To compare the cluster composition between the treatment conditions, as well as to the in vivo IPA dataset, we integrated all three together (Fig. 4, F to I, and fig. S5D). We identified cells in both the Ascl1-only in vitro and IPA-reprogrammed MG in vitro that mapped to the neuron clusters from the IPA in vivo dataset. However, each treatment stimulated one main neuron cluster from MG; in the Ascl1 culture, the neurons most closely resembled bipolar cells (Fig. 4, F and I), while in the IPA cultures, the neurons acquired an RGC-like fate (Fig. 4, G and I), with a minority differentiating into bipolar cells. This differed from our observations in vivo, in which the bipolar cluster was similar in proportion to that of the Ascl1-only sample (Figs. 1D and 4, F and H).

Next, we compared the gene expression profiles of the induced neurons in the IPA and Ascl1-only in vitro datasets. We observed that bipolar genes, such as *Cabp5*, were largely restricted to the Ascl1-only neuron cluster. RGC genes, such as *Grin2a* and *Calb1*, were found only in the IPA neurons (Fig. 4J). To identify unique marker genes expressed in the IPA neurons, we made a subset of all neuron populations in the IPA and Ascl1-only integrated dataset (Fig. 4K) and performed differential gene expression (DGE) analysis (Fig. 4L). We identified a number of RGC genes enriched in the IPA neurons, while bipolar genes were enriched in the Ascl1 neurons. In addition, we found some canonical markers of the RGC lineage that were not induced in vivo, such as *Sncg*, *Nefm*, and *Pou4f1* that were induced in vitro (Fig. 4M). We also failed to detect cells expressing the intrinsic photosensitive RGC marker *Opn4* (fig. S5E). These results collectively suggest that concurrent overexpression of all three IPA factors biases MG-derived neurons to an RGC-like fate and induces more uniform RGC gene expression.

IPA induces neurons with diverse electrical properties

We found previously that Ascl1 can stimulate MG-derived neurons that have physiological characteristics of endogenous retinal neurons, particularly bipolar cells (*4*, *13*). Because IPA treatment leads to a different molecular and morphological neuronal phenotype compared to Ascl1 alone, we characterized the light responses and electrical properties of these cells. We performed patch-clamp electrophysiology on GFP^+ cells in retinal slices and whole mounts after our IPA in vivo regeneration paradigm. We measured responses to current and voltage steps and responses to light stimuli. We plotted the membrane resistance and resting potential of IPA-induced neurons compared to GFP^- MG, endogenous neurons, and neurons from our previous regeneration strategies using Ascl1 or Ascl1:Atoh1 (Fig. 5A) (*4*, *5*, *13*). Glial cells have low membrane resistance, hyperpolarized resting potentials, and little in the way of voltage-activated conductance. Neurons have higher resistance, have less-negative resting potential, and express voltage-activated conductance.

Most of the IPA-induced neurons displayed resting potential and membrane resistance profiles similar to endogenous neurons; however, some of these cells still had glial-like hyperpolarized membrane potentials (Fig. 5A). This is consistent with our IHC and scRNA-seq analysis where a portion of glia does not reprogram after IPA treatment (see Figs. 1 and 3). Figure 5B shows examples of responses to families of current or voltage steps recorded from IPA-treated GFP^+ cells. These cells exhibit a range of characteristics. Some have a neuronal phenotype and appear to express voltage-activated K^+ conductance that limit the extent of depolarization to current steps, while others retain features of glia (Fig. 5B). Six of 16 recorded cells responded to brief light flashes, indicating that they established synaptic connections with other components of the retinal circuitry (Fig. 5C). Some cells responded to current steps by generating action potentials and others generated spiking activity likely representing Ca^{2+} spikes (Fig. 5D). This diversity of the physiological properties is consistent with the phenotypes observed in Figs. 1 and 3. Notably, we did not observe action potentials, a distinct feature of RGCs, in our previous strategies to stimulate MG-derived neurons (*4*, *5*, *13*). Thus, IPA increases the diversity of the electrical properties of the MG-derived neurons, including generating some cells that can produce Na^+ and/or Ca^{2+} action potentials.

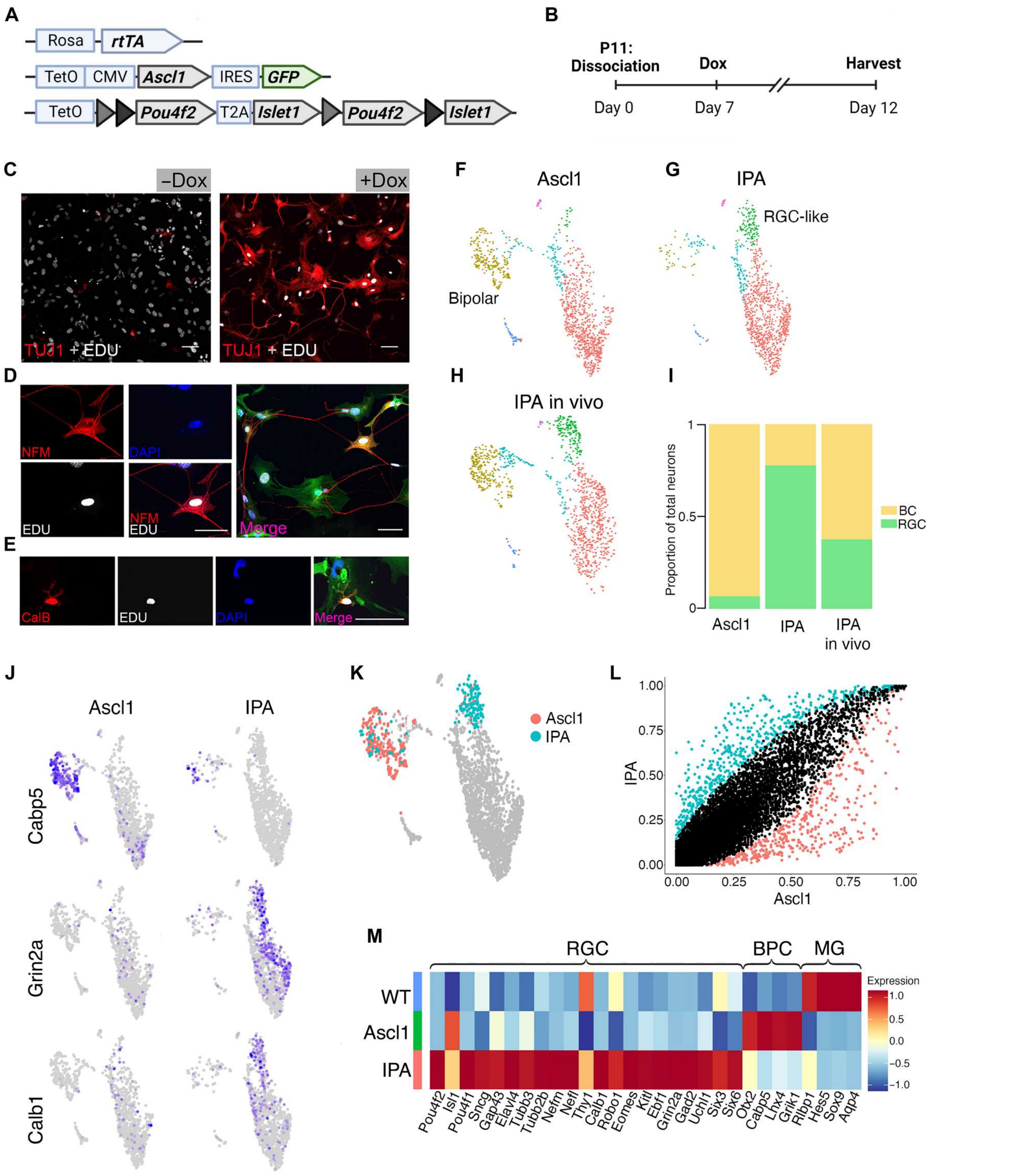

Fig. 4. Islet1 and Pou4f2 coinduction stimulates RGC-like neurons from MG in vitro. (**A**) Schematic of transgenic construct to induce IPA in all primary MG in vitro by doxycycline. (**B**) Paradigm for inducing Ascl1-mediated neurogenesis in vitro. (**C** to **E**) Representative images of EdU$^+$ MG-derived neurons expressing neuronal markers.) (C) EdU$^+$ (white) MG-derived cell expressing Tuj1 (red). (D) MG-derived neuron expressing EdU (white), Neurofilament M (NFM; red), and the GFP transgene reporter (GFP). ((E) MG-derived neuron expressing Calbindin (red) colabeling with EdU (white), DAPI, and GFP. (**F** to **H**) UMAP plots of cultured MG reprogrammed with Ascl1 (F) and IPA (G), integrated with cells from in vivo IPA regeneration model (H) as reference. (**I**) Stacked bar plot showing composition of neuronal clusters in each sample. BC, bipolar cell. (**J**) Feature plots highlighting differentially expressed genes in neuronal clusters of either reprogramming strategy. (**K**) Highlighted cells of the Ascl1 and IPA datasets used for downstream DGE analysis in (**L**). (**M**) Heatmap of genes differentially expressed in either the Ascl1 or IPA condition. WT, untreated cultured MG included for baseline values. Statistics for differential gene analysis: Wilcoxon Mann-Whitney test for significance ($P < 0.05$). Scale bars, 50 μm. Mouse schematic was made with Biorender.com.

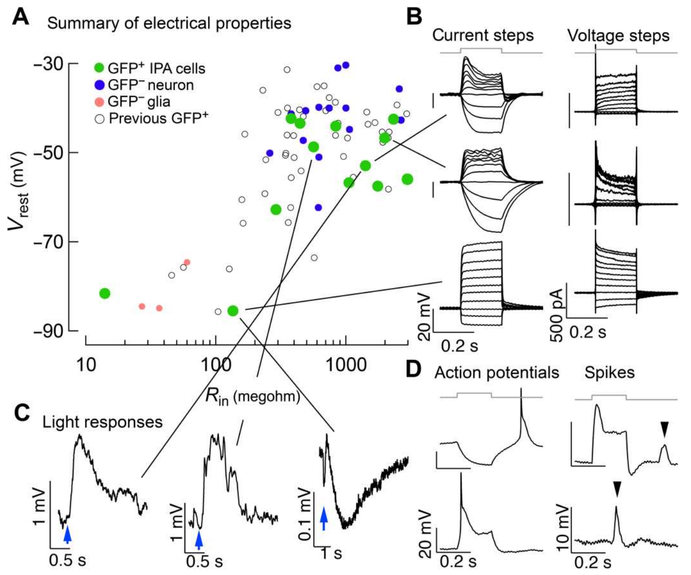

Fig. 5. Physiological profiling of IPA-induced neurons. (**A**) Summary of electrical properties of cells in this study compared to endogenous neurons, endogenous glia, and MG-derived neurons from previous regeneration protocols (*4*, *5*, *13*). Resting potential and input resistance were estimated from current clamp recordings. (**B**) Examples of responses to current (left) and voltage (right) steps for three cells. (**C**) Three examples of cells that responded to light stimulus. (**D**) Examples of cells that displayed action potentials or similar events. The two left panels are responses to hyperpolarizing and depolarizing current steps from a cell that generated apparent Na^+ spikes. The right two panels are responses from a cell that generated smaller discrete events, likely Ca^{2+} spikes.

IPA expression remodels MG chromatin to an imperfect RGC-like fate

We next carried out single-cell assay for transposase-accessible chromatin sequencing (scATAC-seq) on Ascl1 versus IPA-reprogrammed MG to gain a better understanding of the mechanistic differences between these two reprogramming strategies. Mice were treated with the same in vivo retinal regeneration paradigm described in Fig. 3 but were processed for scATAC-seq instead of scRNA-seq. Nuclei (1692) from Ascl1 only and 2451 nuclei from IPA treatment passed our quality control metrics (see Materials and Methods). Single cells from these two treatments were then integrated and plotted as a UMAP to identify cell types (Fig. 6, A to C). Cell type clusters were identified by the pattern of accessible chromatin near genes identified with specific retinal cell types. Coverage plots show representative peaks for the groups we identified: MG (*Rlbp1*$^+$), neurogenic transition (*Islet1*$^+$), MG-derived bipolar cells (*Crx*$^+$), induced RGC-like cells (*Pou4f2*$^+$), and photoreceptors (*Arr3*$^+$) (Fig. 6D). When the UMAP is split between the two treatment groups, it is clear that, while a large cluster of cells retain a MG phenotype, both the Ascl1 and the IPA conditions lead to MG-derived bipolar neurons (Fig. 6, B and C). However, the IPA treatment induces a unique cluster of cells that have accessible chromatin that most closely resemble RGCs (green cluster) (Fig. 6C). Consistent with accessibility patterns, the motif for Otx2 is enriched in the bipolar cluster, while the Pou4f2 motif is highly represented in the accessible chromatin of the IPA-unique cluster (Fig. 6F). This is consistent with our scRNA-seq findings and demonstrates that these MG-derived neurons have patterns of cis-regulatory regions consistent with their transcriptomic identity.

To determine how closely the chromatin of MG-derived RGCs resembles that of normal, developing RGCs, we generated a scATAC library from the E14 developing mouse retina to compare with the MG-derived RGCs (fig. S6, A to F). The scatterplot in Fig. 6G shows that there is significant correlation between the accessible peaks in E14 RGCs and the MG-derived neurons; however, there are clearly many regions that are differentially accessible between cell types (Fig. 6G). When we assayed for GO enrichment to understand the types of genes that are more highly represented in nearby accessible chromatin in E14 RGCs than in MG-derived RGCs, many of the top terms were related to axon growth (Fig. 6H and table S2). This is consistent with the fact that many MG-derived RGCs do not extend long axons.

The current model of retinal development suggests that a cascade of TFs is sequentially activated when cells transition through multipotent progenitors, to neurogenic precursors, to their ultimate neuronal cell fate (*27*). Therefore, we sought to examine whether neurogenesis induced in MG recapitulates developmental cascades at the chromatin level. We used pseudotime to order cells in a projected lineage from the progenitor cells to RGCs in the E14 retina and compared this with a similar analysis of MG-derived RGCs and then analyzed the motif enrichment in accessible chromatin over pseudotime in a "cascade plot" (Fig. 6, I and J) (*28*). This analysis reveals key differences in the process of RGC development versus RGC production from MG. Normal development of RGCs shows down-regulation of progenitor TF motifs (Rax and Sox2) in accessible regions followed by a transient increase in regions with bHLH motifs (Atoh7), and then finally induction of regions with mature RGC TF motifs (Pou4f1/2/3, Ebf1, and Onecut). This has recently been described in both mouse and human (*28*, *29*). By contrast, the sequential changes in motif representation in accessible chromatin in MG reprogrammed to generate neurons with IPA show clear differences from normal development. Although there is a reduction in progenitor gene motifs as cells acquire an RGC-like identity, the progenitor TFs are apparently never fully down-regulated, because their motifs persist in the accessible regions of MG-derived RGCs (Fig. 6J). In addition, although MG-derived RGCs show an increase in accessible bHLH motifs and a robust increase in Pou4f1/2/3 motif representation in accessible chromatin, the TFs that are presumably downstream of Pou4f1/3, such as Onecut and Ebf, are not sequentially activated (Fig. 6J). This epigenomic analysis suggests that RGC-like generation is imperfect from IPA-treated MG, in part because of the maintenance of accessibility at glial and progenitor regulatory regions. Consistent with this notion, the progenitor/MG marker Sox2 is still detectable by immunofluorescence 3 weeks after IPA reprogramming in GFP$^+$ cells with neuronal morphology (Fig. 6K).

Atoh1 can improve the ability of IPA to induce RGC-like cells from MG

The scRNA and scATAC-seq analysis revealed that, although IPA-induced neurons resembled RGCs, these cells lack some features of mature RGCs. One hypothesis for why this is the case is the persistence of glial and progenitor genes and chromatin accessibility (i.e., Sox, Rax, Vsx2, etc.) in the MG-derived neurons. Recently, we have reported that the Atoh class of TFs, when combined with Ascl1, can potently stimulate neurogenesis (*13*); the combination of

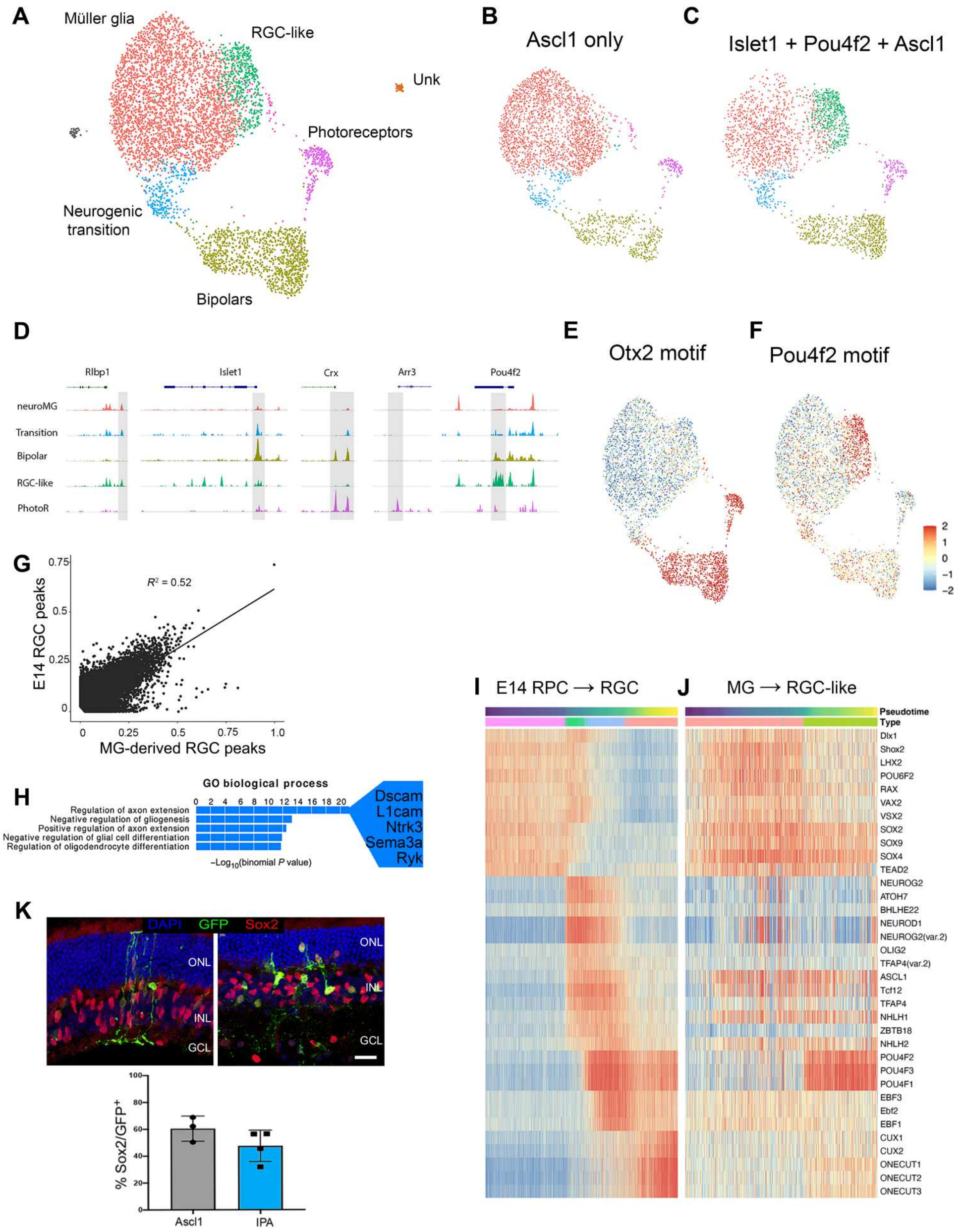

Fig. 6. scATAC reveals MG remodel chromatin to an RGC-like state in response to IPA treatment. (**A**) Combined UMAP of GFP$^+$ sorted MG and their progeny from the in vivo regeneration paradigm with Ascl1-only (**B**) or IPA treatment (**C**). (**D**) Coverage plots for known marker genes used to identify clusters. (**E** and **F**) chromVAR scores of Otx2 and Pou4f2 to highlight differential accessibility of their respective motifs. (**G**) Scatterplot comparing accessible motifs in E14 RGCs versus IPA-induced RGCs. (**H**) Top "GO biological process" results for peaks specific to E14 RGCs compared to IPA-derived RGC-like neurons. (**I**) Heatmap of the chromVAR activity scores of the top variable motifs for TFs found on the pseudotime lineage of E14 progenitor cells to RGCs. (**J**) Heatmap of the same chromVAR activity scores of the E14 motifs plotted on pseudotime from MG to RGC-like neuron after IPA treatment. (**K**) Retinal sections showing GFP$^+$ MG-derived cells costained with the MG nuclei marker Sox2 (red) and quantification of GFP$^+$ cells expressing Sox2. Scale bars, 50 μm.

Ascl1:Atoh1 results in nearly 80% of transgene-expressing MG acquiring a neural identity. Therefore, we tested whether combining Atoh1 with IPA reprogramming could improve the regeneration of RGCs from MG in vivo.

We crossed mice containing a tetracycline-inducible Atoh1 to the IPA strain and carried out the regeneration paradigm as described in Fig. 1; we then performed scRNA-seq and IHC analysis of MG progeny as described above (Fig. 7, A and B). Consistent with our previous findings using Atoh1, the expression of IPA and Atoh1 in MG caused most of the MG progeny to acquire a neuronal identity (*13*). IHC revealed that most GFP$^+$ MG-derived neurons expressed the ganglion/amacrine marker HuC/D and lacked expression of the bipolar marker Otx2 (Fig. 7C). Adding Atoh1 to the combination of IPA factors also bypasses the requirement for retinal injury to induce MG neurogenesis (fig. S7, A to D). We next performed scRNA-seq on regenerated cells treated from the

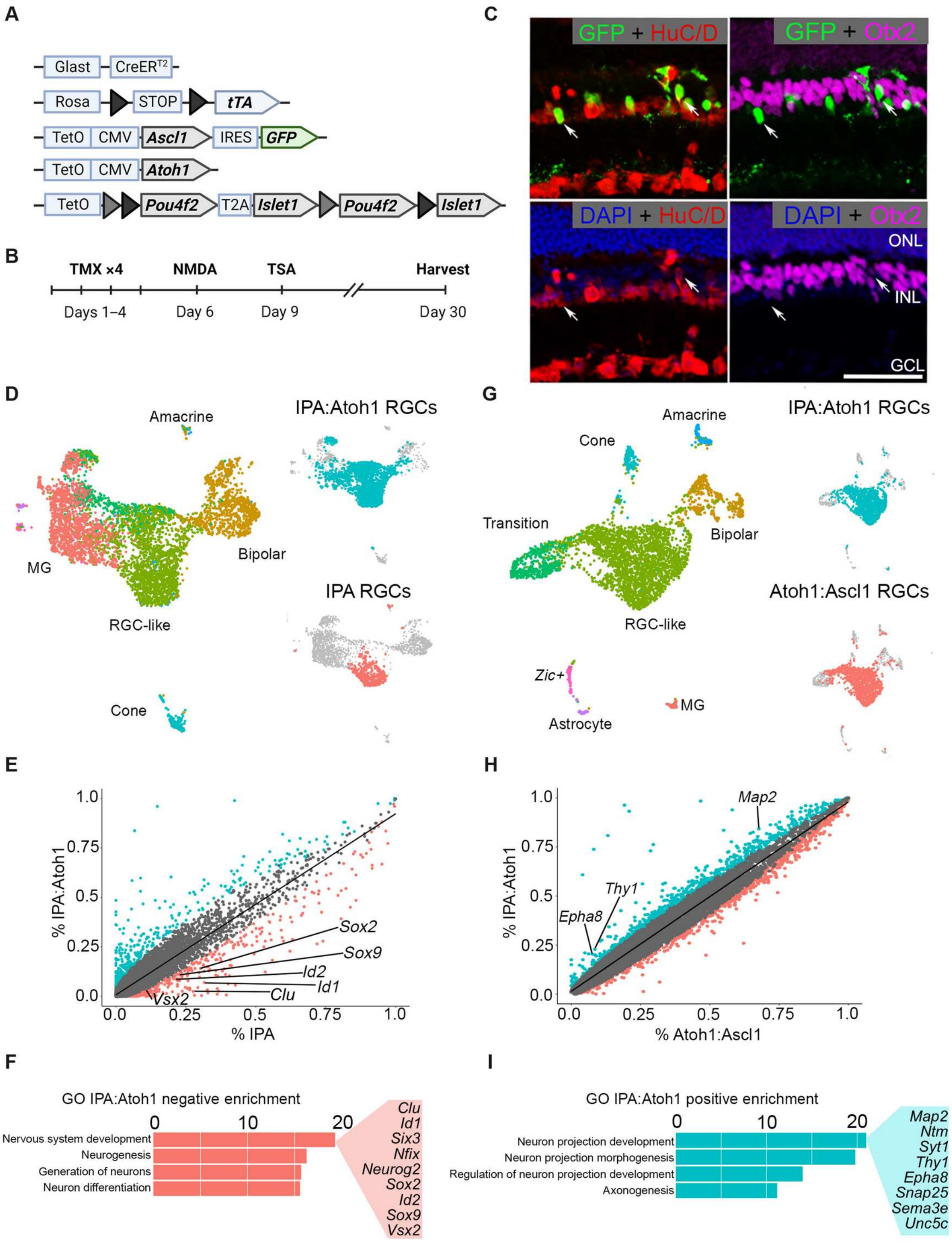

Fig. 7. The addition of Atoh1 to the IPA paradigm facilitates transition from a progenitor state to a differentiated neuron. (**A**) Schematic of transgenic construct to express IPA with Atoh1 in MG. (**B**) Regeneration paradigm for inducing IPA:Atoh1 expression in MG in the damaged retina. (**C**) Representative immunofluorescence images of regenerated neurons from IPA:Atoh1 mice demonstrating MG-derived neurons (GFP^+) are HuC/D^+ (red) and not $Otx2^+$ (purple). (**D**) Integrated UMAP of FACS-sorted MG-derived cells after regeneration paradigm with either IPA:Atoh1 or IPA-only overexpression. Highlighted in either blue or red are the RGC-like cells from each dataset that were subsetted for further comparative analysis. (**E**) Scatterplot highlighting differentially expressed genes between the RGC-like cells of the IPA:Atoh1 (blue) and IPA-only (red) regeneration paradigms. (**F**) GO analysis revealed that neurodevelopmental terms containing many retinal progenitor genes were down-regulated in the IPA:Atoh1 dataset versus IPA only. (**G**) Integrated UMAP of IPA:Atoh1 data as described above with previously generated Ascl1:Atoh1 dataset (*13*). Blue or red highlighting denotes RGC-like cells from each dataset compared in further analysis. (**H**) Scatterplot highlighting differentially expressed genes between the RGC-like cells of the IPA:Atoh1 and Ascl1:Atoh1 regeneration paradigms. (**I**) Bar plot of GO terms relating to neurite outgrowth enriched in the IPA:Atoh1 data. Known neuron projection genes listed are up-regulated with IPA:Atoh1 versus Ascl1:Atoh1. Mouse schematic was made with Biorender.com.

IPA:Atoh1 condition and integrated them with cells from the IPA-only treatment group to determine whether the addition of Atoh1 may improve RGC generation after retinal damage (Fig. 7D). This analysis revealed that MG-derived neurons from the IPA:Atoh1 mice were most similar to the MG-derived RGCs from the IPA condition, while a smaller proportion of bipolar, cone, and amacrine cells were also present (Fig. 7D and fig. S7, E and F).

To assess whether Atoh1 overexpression reduces the progenitor signature of MG-derived neurons after IPA expression, we formed a subset of the RGC-like cells from each dataset for comparison. DGE analysis showed that, in the IPA:Atoh condition, the RGC-like cells showed a greater decrease in expression of progenitor and glial genes than similar cells from the IPA only condition (Fig. 7E). This was further confirmed with GO analysis, where IPA:Atoh1 reduced genes associated with neural progenitors compared to the IPA-only condition (Fig. 7F), consistent with the hypothesis that Atoh1 promotes maturation of the RGC-like cells.

We have previously shown Ascl1:Atoh1 generates immature RGC-like cells from MG (*13*). We hypothesized that the addition of IPA factors to Atoh1 might induce markers of more mature RGCs. We compared IPA:Atoh1 with previously generated Ascl1:Atoh1 datasets and found a similar composition of cell types (Fig. 7G and fig. S7, E to H). However, we found that the addition of IPA to Atoh1 increased RGC-like cells by 15% and resulted in a corresponding decrease in bipolar and progenitor-like cells (fig. S7, G and H). DGE analysis revealed that the addition of IPA to Ascl1:Atoh1 leads to an enrichment of RGC genes such as *Map2* and *Thy1* (Fig. 7H). Furthermore, we found that the top GO terms enriched in the IPA:Atoh1 were related to neuron projection development and axonogenesis (Fig. 7I), with many genes specifically related to axon guidance (e.g., *Epha8* and *Sema3e*) and synaptogenesis (e.g., *Syt1* and *Snap25*).

DISCUSSION

Nonmammalian adult vertebrates can regenerate neurons in many regions of their central nervous system (CNS). For example, after tail amputation in larval frogs and some adult urodeles, the radial glial cells of the spinal cord acquire a pattern of gene expression similar to neuronal precursors and go on to proliferate and regenerate an apparently normal spinal cord (*30*). Similarly, in the retina and brain of zebrafish, glia respond to injury by activating a progenitor-like gene expression program of TFs (*31*). These glia-derived progenitor cells undergo multiple rounds of mitotic cell divisions, and the progeny differentiate into the range of neuron types that can restore function in the brain and retina (*32*).

Reexpressing developmentally active TFs in adult mammalian glia can trigger a regenerative process in these cells that, in many ways, resembles what is found in fish and amphibians. For example, after retinal injury, the transgenic overexpression of the proneural TF Ascl1, combined with histone deacetylase inhibition, can stimulate MG to acquire a progenitor-like state with the capacity of generating bipolar neurons (*4*). The MG-derived neurons differentiate to the point that they make synapses with the surrounding neuronal circuitry and respond to light. In addition to lineage tracing the neurons to validate their glial derivation, we have used EdU labeling to show their adult origin and have profiled the cells using scRNA-seq, and scATAC-seq, to observe intermediate states between glial progenitor and regenerated neurons (*5*, *12*, *13*). Together, this validates that TFs can reprogram glia in the adult CNS to generate neurons.

Although Ascl1 induces MG to adopt many features of retinal progenitors, including proliferative neurogenesis and a transcriptional and epigenetic landscape similar to developmental progenitors, not all developmentally appropriate Ascl1 targets are induced in MG-derived progenitor cells, and the neuronal output from Ascl1 MG is restricted to primarily bipolar neurons (*5*). Thus, we reasoned that additional TFs might be required to properly steer MG-derived progenitors to specific types of neurons. This is particularly important for endogenous regeneration strategies because most blinding diseases are the result of deficits in a particular neuronal subtype. For example, glaucoma is primarily caused by the death of RGCs.

RGCs are generated during development by a cascade of TFs, characterized by the initial expression of Atoh7 and the downstream expression of additional TFs, such as Pou4f1/2 and Islet1 (*33*). Atoh7 is necessary for proper RGC fate by inducing these downstream stabilizing TFs (*34*–*36*). Two of these downstream TFs, Pou4f2 and Islet1, are required for proper RGC fate specification (*18*, *37*, *38*), and ectopic expression of Pou4f2 and Islet1 in the Atoh7 null retina is sufficient to rescue the RGC fate (*19*). By taking advantage of the wealth of knowledge of normal transcriptional regulation of the RGC fate, we were able to test whether members of this TF cascade can reinitiate the genesis of these cells from the Ascl1-induced MG-derived progenitors.

We report here that the RGC fate–inducing factors, Islet1 and Pou4f2, along with Ascl1, can induce MG neurogenesis toward an RGC-like fate. Using IHC, we show that the MG-derived neurons, from IPA-expressing MG, express protein markers and morphological features of RGCs. At the physiological level, these IPA-induced, MG-derived neurons display neuronal resting membrane potentials and a subset generated voltage-gated action potentials, consistent with an RGC-like fate. Last, our molecular analysis using scRNA-seq and scATAC-seq show that, at the transcriptomic and epigenetic levels, IPA-induced MG-derived neurons most closely resemble immature RGC cells.

Although the expression of the IPA transgenes in MG collectively reprograms a subset of the cells to an RGC-like fate, this combination does not activate the full complement of TFs required for mature RGCs. We find both in the scRNA-seq and in the scATAC-seq datasets that some mature RGC genes fail to be induced; instead, the MG-derived neurons retain expression of some progenitor/glial genes and a resulting chromatin landscape intermediate between progenitors and RGCs. The progenitor state in MG can be repressed by the expression of Atoh1 in addition to IPA, consistent with the role for atonal TFs in promoting neuronal differentiation, and this allows further differentiation of the MG-derived RGCs. Together, our results show that the ectopic expression of developmental TFs that are integral to a cell type–specific trajectory can reprogram MG toward that same trajectory. This suggests an overall approach where the complementary and sequential actions of TFs in development of specific neuronal types can be used to regenerate more mature neurons. It remains to be seen whether expression of TFs in their normal developmental sequence will provide a more effective strategy for reprogramming.

It is interesting that the RGC-like neurons derived from MG are most frequently found in the inner nuclear layer (INL), instead of the ganglion cell layer (GCL). Although a small percentage of RGCs

is normally found in the INL in mice, most migrate to the GCL. It is possible that the inner plexiform layer provides a barrier to migration of the RGC-like cells, or alternatively, they may lack the cues for appropriate migration. Although the scRNA-seq data show MG-derived RGC-like cells express many RGC genes, they may lack some critical migration program. Nevertheless, some of these cells connect with the existing neural circuitry and respond to light, and it may be that appropriate connectivity can be established without proper somal location.

Although we also did not observe robust axonal outgrowth directed to the optic nerve, we find that many genes important for axon growth and guidance are expressed in the RGC-like cells. It is possible that some key guidance factors are not expressed in these cells or, alternatively, that the adult retinal environment no longer expresses the guidance factors needed to direct axons to the optic nerve head. RGC transplant experiments have found that, in some cases, the RGC axons project ectopically, suggesting that the microenvironment of the adult retina may not fully reflect that of the developing retina; future studies will be needed to better understand the interplay between cell-autonomous factors and nonautonomous factors in regeneration.

Over the past decade, a number of reports have suggested that other developmental TFs such as Sox2, Pax6, NeuroD1, Neurog2, and Ascl1 can stimulate neurogenesis from glia both in vitro and in the brain (*39*). However, the interpretation of many of these reports has been clouded by the finding that lineage tracing new neurons is unreliable using existing Adeno-Associated Virus (AAV) paradigms and often leads to endogenous neurons being misidentified as glial derived (*40*, *41*). Nevertheless, some of the earlier studies that used alternative methods to trace glial-derived neurons were able to show that reprogramming of astrocytes to neurons is possible, and proneural TFs, including Ascl1, are among the neurogenic stimulants (*42*–*45*). Thus, it is possible that the strategy of combining TFs from a specific neuronal lineage, as we have used for the retina, will have applicability throughout the CNS.

Overall, this study provides a proof of concept that TFs used to specify cell fate in development can be harnessed to initiate similar fate acquisition from reprogrammed glia cells in the adult CNS. The re-engineering process of cell fate acquisition during endogenous regeneration strategies will be key in guiding neural replacement appropriate for specific neurodegenerative diseases.

MATERIALS AND METHODS

Primary cell culture

Retinas from tetO-Ascl1-GFP or tetO-Pou4f2-Islet1-Ascl1-GFP mice of both sexes were harvested at Postnatal Day 12 (P12) for MG cultures. For dissociation, the retinas were incubated in a solution of papain and deoxyribonuclease (DNase) (Worthington) for 10 min at 37°C, followed by trituration. To stop the reaction, an equal volume of ovomucoid (Worthington) was added. Cells were then spun at 4°C at 300*g* for 10 min and resuspended in growth medium consisting of Neurobasal (Gibco), 10% fetal bovine serum (FBS) (Clontech), N2 (Invitrogen), 1 mM L-glutamine (Invitrogen), 1% penicillin-streptomycin (Invitrogen), and mouse epidermal growth factor (100 ng/ml) (R&D Systems). Cells were plated at a density of two retinas per 10cm^2 in a six-well dish and incubated at 37°C. The medium was changed every 2 days until confluent (~7 days). At confluence, cells were passaged with TrypLE (Gibco); resuspended in a freezing medium consisting of 50% growth medium, 40% FBS, and 10% dimethyl sulfoxide (DMSO); and stored at least 1 day in liquid NO_2 for at least 24 hours. After thawing, cells were grown in FBS-reduced growth medium (1%) and treated with doxycycline to induce tetO-mediate genes.

Animals

All animals were treated and housed with University of Washington Institutional Animal Care and Use Committee approved protocols. The (i) *Glast-CreER:LNL-tTA:tetO-mAscl1-ires-GFP*, (ii) *Glast-CreER:LNL-tTA:tetO-P&I:tetO-mAscl1-ires-GFP*, (iii) *Glast-CreER:LNL-tTA:tetO-Atoh1:tetO-P&I:tetO-mAscl1-ires-GFP* mice, (iv) rtTa:tetO-Ascl1-ires-GFP, and (v) rtTa:tetO-P7I:tetO-Ascl1-ires-GFP are from mixed backgrounds of C57BL/6 and B6SJF1. The Glast-CreER, LNLtTA, and rtTa mice are from the Jackson Laboratory. The tetO-mAscl1-GFP mice were a gift from M. Nakafuku (University of Cincinnati), the tetO-Atoh1 mice were a gift from P. Chen (Emory University), and the tetO-P&I mice were a gift from X. Mu (University of Buffalo). Males and females were both used in experiments at equal frequencies. All in vivo experiments were performed on adult mice that were over 40 days old.

Immunohistochemistry

After CO_2-mediated euthanasia, corneas were removed and eye globes were fixed for 30 min in 4% paraformaldehyde (PFA) in phosphate-buffered saline (PBS). Fixed eyes were then incubated overnight in 30% sucrose at 4°C. Retinas were then frozen in optimal cutting temperature compound (O.T.C.) and cryosectioned at 18 μm. For immunostaining, sections were washed two times for 10 min in PBS and then incubated in primary antibody in 0.5% Triton X-10 and PBS overnight. Slides were then washed in PBS and incubated in secondary antibodies for 1 hour. Last, slides were washed again in PBS and coverslipped with Fluoromount-G (SouthernBiotech). See table S1 for antibody and concentration information.

Cultured MG were plated on glass coverslips with FBS-reduced growth medium (described above). To activate the transgenes, doxycycline was added at a concentration of 3 μg/ml every 24 hours for a period of 5 days. The coverslips were washed with PBS and then fixed with 4% PFA for 10 min at room temperature (RT), followed by 3 × 5 min of washes with PBS. Primary antibody incubation was done in 0.5% Triton X-10 and 10% normal horse serum (NHS) in PBS overnight at 4°C. Coverslips were washed in PBS and then click chemistry was used to label EdU (Click-iT EdU Assay, Invitrogen), followed by incubation with secondary antibodies and 4′,6-diamidino-2-phenylindole at RT for 1 hour. After staining, the coverslips were washed three more times and placed onto slides with Fluoromount-G (SouthernBiotech).

Fluorescence-activated cell sorting

Following euthanasia, retinas were dissociated into single cells as described for cell culture; after pelleting at 300*g* at 4°C, cells resuspended in Neurobasal solution and passed through a 35-μm filter. Using a BD FACSAria III cell sorter (BD Bioscience), FACS was performed on GFP$^+$ cells.

Injections
Intravitreal injections were performed with a 32-G Hamilton syringe on mice anesthetized with isoflurane. Injections of NMDA were done in a volume of 1 μl at a concentration of 100 mM in PBS. TSA (Sigma-Aldrich) was administered via intravitreal injections in DMSO at a concentration of 1 μg/μl. Intraperitoneal injections of tamoxifen (1.5 mg per 100 μl of corn oil) were administered to adult mice for four consecutive days to induce expression of the tetO-mAscl1-ires-GFP, the tetO-P&I, and the tetO-Atoh1 gene.

Microscopy/cell counts
Images were taken on a Zeiss LSM880 confocal microscope. For quantification of cell counts, a minimum of four images per retina with a 20× objective were taken at the same magnification.

Electrophysiology
Recordings were performed identical to our previous reports (*4*, *5*). Mice were dark-adapted before recordings. After euthanasia, retinas were sliced into 200-μm slices for recording. Tissue recordings were performed in Ames medium at 32° C and oxygenated with 95% O_2/5% CO_2. GFP^+ cells were targeted for recording using video differential interference contrast with infrared light and confocal microscopy. Light responses were measured under infrared conditions, and the tissue was exposed to full-field illumination via blue and green light-emitting diodes. Recordings were performed using pulled glass pipettes and filled with solution containing the following: 123 mM K-aspartate, 10 mM Hepes, 1 mM $MgCl_2$, 10 mM KCl, 1 mM $CaCl_2$, 2 mM EGTA, 0.5 mM tris–guanosine triphosphate, 4 mM MG–adenosine triphosphate, and 0.1 mM Alexa Fluor 695 hydrazide.

Single-cell RNA library construction
For in vivo datasets, following FACS purification of GFP^+ MG, cells were centrifuged at 300*g* at 4°C and resuspended at a concentration of 1000 cells/μl. Library construction was done using 10x Genomics 3′ single-cell RNA V3 or V3.1 sequencing kits as described by the manufacturer. Cells were encapsulated in gel beads and in given unique barcodes using the 10x Chromium controller and 10x Genomics chip type G. Libraries were multiplexed using single and dual index kits (10x Genomics).

For in vitro experiments, MG cultures from three to five mice were used. Following 5 days of doxycycline treatment, cells were dissociated using a mix of Accutase (Sigma-Aldrich, Saint-Louis, MO) and DNAse (Worhington, Lakewood, NJ) for 2 min and then spun down at 400 relative centrifugal force for 7 min at 4°C. The pellet was resuspended in culture medium to reach a targeted concentration of 1000 cells/μl. Cells were passed through a strainer and loaded into the 10x Genomics Chromium Single Cell chip G following the protocol of Chromium Single Cell 3′ Reagents Kits v3.1 (10x Genomics, Pleasanton, CA).

Single-cell RNA sequencing, mapping, and data analysis
Libraries were sequenced using an Illumina NextSeq 500, in most cases using multiplexed libraries using high-output 150 kits. Data were demultiplexed and aligned to the mm10 genome using CellRanger version 3.0. Filtered output files were further analyzed in R using Seurat version ≥3.0, ggplot2, data.table, dplyr, tidyr, and other commonly used R packages. Low-quality cells (identified as having low read depth or high mitochondrial content; >10%) were removed from datasets. Microglia, as well as any astrocytes or endothelial cells present (for in vitro experiments), were identified and removed from the data before downstream analysis. In addition, before analysis, gene expression data were normalized and scaled, and cells were clustered using principal components analysis and UMAP, using the tools available in the Seurat R package version ≥3.0. Comparisons between datasets were done by canonical correlation analysis as described by the Satija laboratory vignette (*46*) (https://satijalab.org/seurat/archive/v3.0/integration.html).

Integration with development data
For comparison to developing retina, data were first downloaded from Gene Expression Omnibus (GEO) (*21*). The label transfer was carried out in Seurat using a reference dataset composed of 432 randomly sampled cells of each major cell class in the developing retina dataset (Rod, Cone, Bipolar, Amacrine, Horizontal, Retinal Ganglion, and Müller glia), for a total of 3024 cells, as identified by canonical markers. Reads were downsampled to a common average depth before analysis. In addition, the IPA neurons were integrated directly with a subset composed only of E14 cells from the development dataset.

Single-cell ATAC sequencing
The Cellranger ATAC pipeline (2.0.0) was used to preprocess the data resulting from sequencing (*47*). First, "cellranger-atac mkfastq" was used to convert BCL files to fastqs and demultiplex reads. Next, "cellranger-atac count" was run to map Tn5 sites to mm10 (mouse genome), remove duplicate reads, and remove background cells. This returned peak by cell matrices and barcoded fragment files that were loaded into Signac (*48*), an R (4.0.4) (R core team, 2021) package. Macs2 was then run on the Signac object and barcoded fragment files to call peaks using Signac's "CallPeaks" function (*49*). Fragments were mapped to the peaks called by Macs2 and assigned to cells using Signac's "FeatureMatrix" function. Further quality control (QC) metrics were measured in Signac using the "NucleosomeSignal" and "TSSEnrichment" functions. Cells who were outliers in the QC metric categories were removed as per Signac's standard processing guidelines. Latent semantic indexing (LSI) was performed in Signac using the "RunTFIDF" and "RunSVD" functions. Signac's "DepthCor" was used to identify LSI dimensions that were highly correlated with read depth; these LSI dimensions were excluded from downstream analysis. Signac/Seurat's "RunUMAP" function was run to compute the UMAP embedding. To identify clusters, Signac/Seurat's "FindClusters" was then run at varying resolutions. Clusters were assigned to known retinal cell types by inspecting Tn5 insertions within 100 kb of known marker genes using Signac's "CoveragePlot" and further supported using chromVAR scores for known lineage-specific TFs. Clusters of the same type were grouped for visualization purposes. Vertebrate motifs were acquired from the Jaspar 2020 database. Signac's "AddMotifs" function was used to map these motifs to peaks within the Signac object. Signac's "RunChromvar" function was used to calculate motif accessibility *z* score across all cells.

Dataset integration
Before integrating Signac objects, we first ran all previous computational steps on each sample independently. Next, we created a shared peak set for all objects that were to be integrated using

BEDOPS (-m) (*50*). Signac's FeatureMatrix function was run on each sample with the merged peak set to put all samples in the same feature space. Samples were next downsampled to the same average read depth using DropletUtils "downsampleMatrix" function. Samples were merged using Signac/Seurat's "merge" function, and standard Signac normalization, dimensional reduction, clustering, and visualization were performed on the merged object as described above. The object was then split by samples, and the samples were integrated together. Anchors between samples were calculated using "FindIntegrationAnchors," and an integrated embedding space was then calculated using Signac's "IntegrateEmbeddings" function. UMAP and clustering for the integrated object were performed as previously described.

Pseudotime analysis

To calculate psuedotime and identify trajectory branches, samples were loaded into Monocle 3 (*51*) using SeuratWrapper's "as.cell_data_set" function. Clustering was performed and partitions were defined using Monocle 3's "cluster_cells." Monocle 3's "learn_graph" was then run to define the principal trajectory graph. The pseudotime root was placed in the progenitor-like or MG-like cluster using Monocle 3's "order_cells." Branches were selected using Monocle 3's "choose_graph_segments." Pseudotime and branch data were transferred back to the Seurat object for later analysis.

Scatterplots

To create the scatterplots, Signac/Seurat's "FindMarkers" function was run between cell groups of interest, using the top 25% most accessible peaks in those cell groups. A scatterplot was then made showing the percent of cells in each group that had accessibility of each peak. Peaks with an average log base twofold change greater than 0.05 were selected for each group. For GO analysis, these peak sets were then loaded into GREAT (Genome Regions Enrichment of Annotations Tool) (*52*) or analyzed with gprofiler2 package in R for scRNA-seq data.

Cascade heatmaps

To construct the Cascade heatmaps, motif names were translated to mouse gene names using R and biomart's "getLDS" function. Pseudotime lineage branches for the RGCs were subset from the RNA and ATAC data from E14 samples. Variable motifs were identified by running chromVAR's "addGCBias," "getBackgroundPeaks," "computeDeviations," and "computeVariability" functions. Peaks with a verbality score greater than 1.2 were kept for further analysis. This motif list was then subset again; only motifs corresponding to TFs expressed by more than 10% of cells in the RGC branch were kept. Last, conjoined motifs were dropped. Motif enrichment scores for selected motifs were ordered over pseudotime in the E14 RGC branch in the scATAC-seq data by fitting their ChomVAR scores to a third-order polynomial function and ordering motifs by the maximum value of this function within our pseudotime range. To create the heatmaps, chromVAR motif accessibility z scores were plotted in the previously derived order over pseudotime within the RGC lineage. RNA heatmaps were created by plotting the genes whose binding sites were in the final motif list in the same order across pseudotime in the RNA object within RGC lineage. The cascade heatmap for the reprogrammed cells was created by plotting motifs and factors identified in the developmental data.

Supplementary Materials

This PDF file includes:
Figs. S1 to S7
Table S1

Other Supplementary Material for this manuscript includes the following:
Table S2

View/request a protocol for this paper from *Bio-protocol*.

REFERENCES AND NOTES

1. L. Todd, T. A. Reh, Comparative biology of vertebrate retinal regeneration: Restoration of vision through cellular reprogramming. *Cold Spring Harb. Perspect. Biol.* **14**, a040816 (2022).
2. A. Hamon, J. E. Roger, X. J. Yang, M. Perron, Müller glial cell-dependent regeneration of the neural retina: An overview across vertebrate model systems. *Dev. Dyn.* **245**, 727–738 (2016).
3. A. Bringmann, I. Iandiev, T. Pannicke, A. Wurm, M. Hollborn, P. Wiedemann, N. N. Osborne, A. Reichenbach, Cellular signaling and factors involved in Müller cell gliosis: Neuroprotective and detrimental effects. *Prog. Retin. Eye Res.* **28**, 423–451 (2009).
4. N. L. Jorstad, M. S. Wilken, W. N. Grimes, S. G. Wohl, L. S. VandenBosch, T. Yoshimatsu, R. O. Wong, F. Rieke, T. A. Reh, Stimulation of functional neuronal regeneration from Müller glia in adult mice. *Nature* **548**, 103–107 (2017).
5. N. L. Jorstad, M. S. Wilken, L. Todd, C. Finkbeiner, P. Nakamura, N. Radulovich, M. J. Hooper, A. Chitsazan, B. A. Wilkerson, F. Rieke, T. A. Reh, STAT signaling modifies Ascl1 chromatin binding and limits neural regeneration from Muller glia in adult mouse retina. *Cell Rep.* **30**, 2195–2208.e5 (2020).
6. T. Hoang, J. Wang, P. Boyd, F. Wang, C. Santiago, L. Jiang, S. Yoo, M. Lahne, L. J. Todd, M. Jia, C. Saez, C. Keuthan, I. Palazzo, N. Squires, W. A. Campbell, F. Rajaii, T. Parayil, V. Trinh, D. W. Kim, G. Wang, L. J. Campbell, J. Ash, A. J. Fischer, D. R. Hyde, J. Qian, S. Blackshaw, Gene regulatory networks controlling vertebrate retinal regeneration. *Science* **370**, eabb8598 (2020).
7. E. M. Rueda, B. M. Hall, M. C. Hill, P. G. Swinton, X. Tong, J. F. Martin, R. A. Poché, The Hippo pathway blocks mammalian retinal Müller glial cell reprogramming. *Cell Rep.* **27**, 1637–1649.e6 (2019).
8. K. Yao, S. Qiu, L. Tian, W. D. Snider, J. G. Flannery, D. V. Schaffer, B. Chen, Wnt regulates proliferation and neurogenic potential of Müller glial cells via a Lin28/let-7 miRNA-dependent pathway in adult mammalian retinas. *Cell Rep.* **17**, 165–178 (2016).
9. K. Yao, S. Qiu, Y. V. Wang, S. J. H. Park, E. J. Mohns, B. Mehta, X. Liu, B. Chang, D. Zenisek, M. C. Crair, J. B. Demb, B. Chen, Restoration of vision after de novo genesis of rod photoreceptors in mammalian retinas. *Nature* **560**, 484–488 (2018).
10. J. Pollak, M. S. Wilken, Y. Ueki, K. E. Cox, J. M. Sullivan, R. J. Taylor, E. M. Levine, T. A. Reh, ASCL1 reprograms mouse Muller glia into neurogenic retinal progenitors. *Development* **140**, 2619–2631 (2013).
11. Y. Ueki, M. S. Wilken, K. E. Cox, L. Chipman, N. Jorstad, K. Sternhagen, M. Simic, K. Ullom, M. Nakafuku, T. A. Reh, Transgenic expression of the proneural transcription factor Ascl1 in Müller glia stimulates retinal regeneration in young mice. *Proc. Natl. Acad. Sci. U.S.A.* **112**, 13717–13722 (2015).
12. L. Todd, C. Finkbeiner, C. K. Wong, M. J. Hooper, T. A. Reh, Microglia suppress Ascl1-induced retinal regeneration in mice. *Cell Rep.* **33**, 108507 (2020).
13. L. Todd, M. J. Hooper, A. K. Haugan, C. Finkbeiner, N. Jorstad, N. Radulovich, C. K. Wong, P. C. Donaldson, W. Jenkins, Q. Chen, F. Rieke, T. A. Reh, Efficient stimulation of retinal regeneration from Müller glia in adult mice using combinations of proneural bHLH transcription factors. *Cell Rep.* **37**, 109857 (2021).
14. C. Powell, E. Cornblath, F. Elsaeidi, J. Wan, D. Goldman, Zebrafish Müller glia-derived progenitors are multipotent, exhibit proliferative biases and regenerate excess neurons. *Sci. Rep.* **6**, 24851 (2016).
15. F. D. D'Orazi, S. C. Suzuki, N. Darling, R. O. Wong, T. Yoshimatsu, Conditional and biased regeneration of cone photoreceptor types in the zebrafish retina. *J. Comp. Neurol.* **528**, 2816–2830 (2020).
16. G. Beykin, A. M. Norcia, V. J. Srinivasan, A. Dubra, J. L. Goldberg, Discovery and clinical translation of novel glaucoma biomarkers. *Prog. Retin. Eye Res.* **80**, 100875 (2021).
17. Y. Elshatory, M. Deng, X. Xie, L. Gan, Expression of the LIM-homeodomain protein Isl1 in the developing and mature mouse retina. *J. Comp. Neurol.* **503**, 182–197 (2007).
18. L. Gan, M. Xiang, L. Zhou, D. S. Wagner, W. H. Klein, J. Nathans, POU domain factor Brn-3b is required for the development of a large set of retinal ganglion cells. *Proc. Natl. Acad. Sci. U.S.A.* **93**, 3920–3925 (1996).

19. F. Wu, T. J. Kaczynski, S. Sethuramanujam, R. Li, V. Jain, M. Slaughter, X. Mu, Two transcription factors, Pou4f2 and Isl1, are sufficient to specify the retinal ganglion cell fate. *Proc. Natl. Acad. Sci. U.S.A.* **112**, E1559–E1568 (2015).
20. A. Usui, Y. Mochizuki, A. Iida, E. Miyauchi, S. Satoh, E. Sock, H. Nakauchi, H. Aburatani, A. Murakami, M. Wegner, S. Watanabe, The early retinal progenitor-expressed gene Sox11 regulates the timing of the differentiation of retinal cells. *Development* **140**, 740–750 (2013).
21. B. S. Clark, G. L. Stein-O'Brien, F. Shiau, G. H. Cannon, E. Davis-Marcisak, T. Sherman, C. P. Santiago, T. V. Hoang, F. Rajaii, R. E. James-Esposito, R. M. Gronostajski, E. J. Fertig, L. A. Goff, S. Blackshaw, Single-cell RNA-seq analysis of retinal development identifies NFI factors as regulating mitotic exit and late-born cell specification. *Neuron* **102**, 1111–1126.e5 (2019).
22. T. Stuart, A. Butler, P. Hoffman, C. Hafemeister, E. Papalexi, W. M. Mauck III, Y. Hao, M. Stoeckius, P. Smibert, R. Satija, Comprehensive integration of single-cell data. *Cell* **177**, 1888–1902.e21 (2019).
23. K. C. Chang, J. Hertz, X. Zhang, X.-L. Jin, P. Shaw, B. A. Derosa, J. Y. Li, P. Venugopalan, D. A. Valenzuela, R. D. Patel, K. R. Russano, S. A. Alshamekh, C. Sun, K. Tenerelli, C. Li, D. Velmeshev, Y. Cheng, T. M. Boyce, A. Dreyfuss, M. S. Uddin, K. J. Muller, D. M. Dykxhoorn, J. L. Goldberg, Novel regulatory mechanisms for the SoxC transcriptional network required for visual pathway development. *J. Neurosci.* **37**, 4967–4981 (2017).
24. Y. Jiang, Q. Ding, X. Xie, R. T. Libby, V. Lefebvre, L. Gan, Transcription factors SOX4 and SOX11 function redundantly to regulate the development of mouse retinal ganglion cells. *J. Biol. Chem.* **288**, 18429–18438 (2013).
25. T. A. Reh, W. Tetzlaff, A. Ertlmaier, H. Zwiers, Developmental study of the expression of B50/GAP-43 in rat retina. *J. Neurobiol.* **24**, 949–958 (1993).
26. Y.-R. Peng, N. M. Tran, A. Krishnaswamy, D. Kostadinov, E. M. Martersteck, J. R. Sanes, Satb1 regulates contactin 5 to pattern dendrites of a mammalian retinal ganglion cell. *Neuron* **95**, 869–883 e866 (2017).
27. E. A. Bassett, V. A. Wallace, Cell fate determination in the vertebrate retina. *Trends Neurosci.* **35**, 565–573 (2012).
28. C. Finkbeiner, I. Ortuño-Lizarán, A. Sridhar, M. Hooper, S. Petter, T. A. Reh, Single-cell ATAC-seq of fetal human retina and stem-cell-derived retinal organoids shows changing chromatin landscapes during cell fate acquisition. *Cell Rep.* **38**, 110294 (2022).
29. P. Lyu, T. Hoang, C. P. Santiago, E. D. Thomas, A. E. Timms, H. Appel, M. Gimmen, N. Le, L. Jiang, D. W. Kim, S. Chen, D. F. Espinoza, A. E. Telger, K. Weir, B. S. Clark, T. J. Cherry, J. Qian, S. Blackshaw, Gene regulatory networks controlling temporal patterning, neurogenesis, and cell-fate specification in mammalian retina. *Cell Rep.* **37**, 109994 (2021).
30. A. D. Kakebeen, A. D. Chitsazan, M. C. Williams, L. M. Saunders, A. E. Wills, Chromatin accessibility dynamics and single cell RNA-Seq reveal new regulators of regeneration in neural progenitors. *eLife* **9**, e52648 (2020).
31. B. W. Lindsey, Z. J. Hall, A. Heuzé, J. S. Joly, V. Tropepe, J. Kaslin, The role of neuro-epithelial-like and radial-glial stem and progenitor cells in development, plasticity, and repair. *Prog. Neurobiol.* **170**, 99–114 (2018).
32. D. Goldman, Müller glial cell reprogramming and retina regeneration. *Nat. Rev. Neurosci.* **15**, 431–442 (2014).
33. F. Wu, J. E. Bard, J. Kann, D. Yergeau, D. Sapkota, Y. Ge, Z. Hu, J. Wang, T. Liu, X. Mu, Single cell transcriptomics reveals lineage trajectory of retinal ganglion cells in wild-type and Atoh7-null retinas. *Nat. Commun.* **12**, 1465 (2021).
34. N. L. Brown, S. Patel, J. Brzezinski, T. Glaser, Math5 is required for retinal ganglion cell and optic nerve formation. *Development* **128**, 2497–2508 (2001).
35. J. N. Kay, K. C. Finger-Baier, T. Roeser, W. Staub, H. Baier, Retinal ganglion cell genesis requires lakritz, a zebrafish atonal homolog. *Neuron* **30**, 725–736 (2001).
36. J. Brodie-Kommit, B. S. Clark, Q. Shi, F. Shiau, D. W. Kim, J. Langel, C. Sheely, P. A. Ruzycki, M. Fries, A. Javed, M. Cayouette, T. Schmidt, T. Badea, T. Glaser, H. Zhao, J. Singer, S. Blackshaw, S. Hattar, Atoh7-independent specification of retinal ganglion cell identity. *Sci. Adv.* **7**, eabe4983 (2021).
37. X. Mu, X. Fu, P. D. Beremand, T. L. Thomas, W. H. Klein, Gene regulation logic in retinal ganglion cell development: Isl1 defines a critical branch distinct from but overlapping with Pou4f2. *Proc. Natl. Acad. Sci. U.S.A.* **105**, 6942–6947 (2008).
38. L. Pan, M. Deng, X. Xie, L. Gan, ISL1 and BRN3B co-regulate the differentiation of murine retinal ganglion cells. *Development* **135**, 1981–1990 (2008).
39. R. Bocchi, G. Masserdotti, M. Götz, Direct neuronal reprogramming: Fast forward from new concepts toward therapeutic approaches. *Neuron* **110**, 366–393 (2022).
40. S. Blackshaw, J. R. Sanes, Turning lead into gold: Reprogramming retinal cells to cure blindness. *J. Clin. Invest.* **131**, e146134 (2021).
41. L.-L. Wang, C. Serrano, X. Zhong, S. Ma, Y. Zou, C.-L. Zhang, Revisiting astrocyte to neuron conversion with lineage tracing in vivo. *Cell* **184**, 5465–5481.e16 (2021).
42. C. Lentini, M. d'Orange, N. Marichal, M.-M. Trottmann, R. Vignoles, L. Foucault, C. Verrier, C. Massera, O. Raineteau, K.-K. Conzelmann, S. Rival-Gervier, A. Depaulis, B. Berninger, C. Heinrich, Reprogramming reactive glia into interneurons reduces chronic seizure activity in a mouse model of mesial temporal lobe epilepsy. *Cell Stem Cell* **28**, 2104–2121.e10 (2021).
43. O. Torper, U. Pfisterer, D. A. Wolf, M. Pereira, S. Lau, J. Jakobsson, A. Björklund, S. Grealish, M. Parmar, Generation of induced neurons via direct conversion in vivo. *Proc. Natl. Acad. Sci. U.S.A.* **110**, 7038–7043 (2013).
44. C. Heinrich, M. Bergami, S. Gascón, A. Lepier, F. Viganò, L. Dimou, B. Sutor, B. Berninger, M. Götz, Sox2-mediated conversion of NG2 glia into induced neurons in the injured adult cerebral cortex. *Stem Cell Rep.* **3**, 1000–1014 (2014).
45. N. Mattugini, R. Bocchi, V. Scheuss, G. L. Russo, O. Torper, C. L. Lao, M. Götz, Inducing different neuronal subtypes from astrocytes in the injured mouse cerebral cortex. *Neuron* **103**, 1086–1095.e5 (2019).
46. A. Butler, P. Hoffman, P. Smibert, E. Papalexi, R. Satija, Integrating single-cell transcriptomic data across different conditions, technologies, and species. *Nat. Biotechnol.* **36**, 411–420 (2018).
47. A. T. Satpathy, J. M. Granja, K. E. Yost, Y. Qi, F. Meschi, G. P. McDermott, B. N. Olsen, M. R. Mumbach, S. E. Pierce, M. R. Corces, P. Shah, J. C. Bell, D. Jhutty, C. M. Nemec, J. Wang, L. Wang, Y. Yin, P. G. Giresi, A. L. S. Chang, G. X. Y. Zheng, W. J. Greenleaf, H. Y. Chang, Massively parallel single-cell chromatin landscapes of human immune cell development and intratumoral T cell exhaustion. *Nat. Biotechnol.* **37**, 925–936 (2019).
48. T. Stuart, A. Srivastava, S. Madad, C. A. Lareau, R. Satija, Single-cell chromatin state analysis with Signac. *Nat. Methods* **18**, 1333–1341 (2021).
49. Y. Zhang, T. Liu, C. A. Meyer, J. Eeckhoute, D. S. Johnson, B. E. Bernstein, C. Nusbaum, R. M. Myers, M. Brown, W. Li, X. S. Liu, Model-based analysis of ChIP-Seq (MACS). *Genome Biol.* **9**, R137 (2018).
50. S. Neph, M. S. Kuehn, A. P. Reynolds, E. Haugen, R. E. Thurman, A. K. Johnson, E. Rynes, M. T. Maurano, J. Vierstra, S. Thomas, R. Sandstrom, R. Humbert, J. A. Stamatoyannopoulos, BEDOPS: High-performance genomic feature operations. *Bioinformatics* **28**, 1919–1920 (2012).
51. J. Cao, M. Spielmann, X. Qiu, X. Huang, D. M. Ibrahim, A. J. Hill, F. Zhang, S. Mundlos, L. Christiansen, F. J. Steemers, C. Trapnell, J. Shendure, The single-cell transcriptional landscape of mammalian organogenesis. *Nature* **566**, 496–502 (2019).
52. C. Y. McLean, D. Bristor, M. Hiller, S. L. Clarke, B. T. Schaar, C. B. Lowe, A. M. Wenger, G. Bejerano, GREAT improves functional interpretation of cis-regulatory regions. *Nat. Biotechnol.* **28**, 495–501 (2010).
53. N. M. Tran, K. Shekhar, I. E. Whitney, A. Jacobi, I. Benhar, G. Hong, W. Yan, X. Adiconis, M. E. Arnold, J. M. Lee, J. Z. Levin, D. Lin, C. Wang, C. M. Lieber, A. Regev, Z. He, J. R. Sanes, Single-cell profiles of retinal ganglion cells differing in resilience to injury reveal neuroprotective genes. *Neuron* **104**, 1039–1055.e12 (2019).

Acknowledgments: We thank the Reh and Bermingham-McDonogh laboratory members for technical advice and critical feedback. We thank the laboratory of X. Mu for providing the tetO-Pou4f2-Islet1 mouse. **Funding:** This work was funded by National Institutes of Health grant NEI R01EY021482 to T.A.R., Gilbert Family Foundation's Vision Restoration Initiative LLC to T.A.R., National Institutes of Health grant NEI K99EY033402 to L.T., Washington Research Foundation WRF fellowship to L.T., International Retina Research Foundation IRRF fellowship to M.J.H., and Mary Gates Undergraduate Scholarship to P.C.D. **Author contributions:** L.T., W.J., and T.A.R. conceptualized the study. L.T., P.C.D., and M.P. performed and analyzed in vivo experiments. W.J. and J.W. performed and analyzed in vitro experiments. L.T., W.J., C.F., M.J.H., and T.A.R. performed single-cell data processing and analysis. N.I. and F.R. conducted electrophysiology experiments. L.T., W.J., F.R., and T.A.R. wrote the original draft of the manuscript. All authors reviewed and edited the manuscript. **Competing interests:** Some of the findings in this report are part of a patent application that has been submitted by the University of Washington: Patent Application 63/362,361 filed 4 January 2022, by inventors L.T., T.A.R., W.J., M.P., and J.W. The authors declare that they have no other competing interests. **Data and materials availability:** All data needed to evaluate the conclusions in the paper are present in the paper and/or the Supplementary Materials. The scRNA-seq and scATAC-seq data generated in this manuscript are deposited at GEO with the accession number GSE211077.

Submitted 26 April 2022
Accepted 26 October 2022
Published 23 November 2022
10.1126/sciadv.abq7219
