# Supplementary material for: Reprogramming Müller glia to regenerate ganglion-like cells in adult mouse retina with developmental transcription factors
Source: Sci Adv. 2022 Nov 23;8(47):eabq7219. doi: 10.1126/sciadv.abq7219 (PMC9683702; doi:10.1126/sciadv.abq7219)
Supplement: Supplementary file 2 — Figs. S1 to S7 Table S1 [file sciadv.abq7219_sm.pdf]

Science Advances
AAAS

Supplementary Materials for

Reprogramming Müller glia to regenerate ganglion-like cells in adult mouse retina with developmental transcription factors

Levi Todd *et al.*

Corresponding author: Thomas A. Reh, tomreh@uw.edu

Sci. Adv. **8**, eabq7219 (2022)
DOI: 10.1126/sciadv.abq7219

The PDF file includes:

Figs. S1 to S7
Table S1
Legend for table S2

Other Supplementary Material for this manuscript includes the following:

Table S2

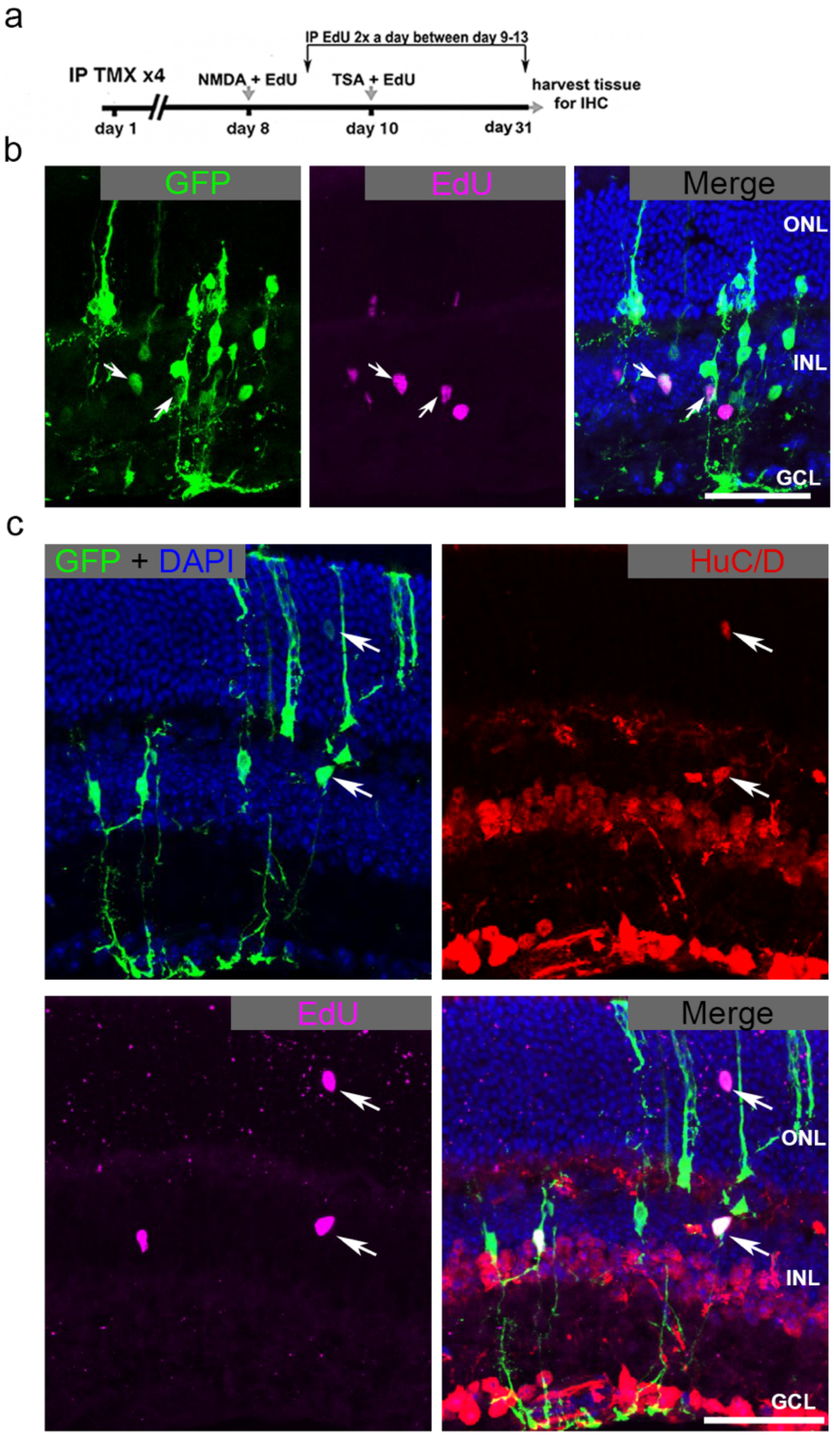

Figure S1: A subset of IPA-derived neurons are derived from proliferating MG. (a) Experimental paradigm to label diving cells during the regeneration experiment described in Figure 1. (b) Representative sections showing MG-derived cells (GFP+) that previously underwent cell division (EdU+). (c) Representative image showing some MG-derived neurons (GFP+/HuC/D+) are the result of proliferating MG (EdU+). Scale bars are 50μm. Abbreviations: ONL, outer nuclear layer, INL, inner nuclear layer, GCL, ganglion cell layer.

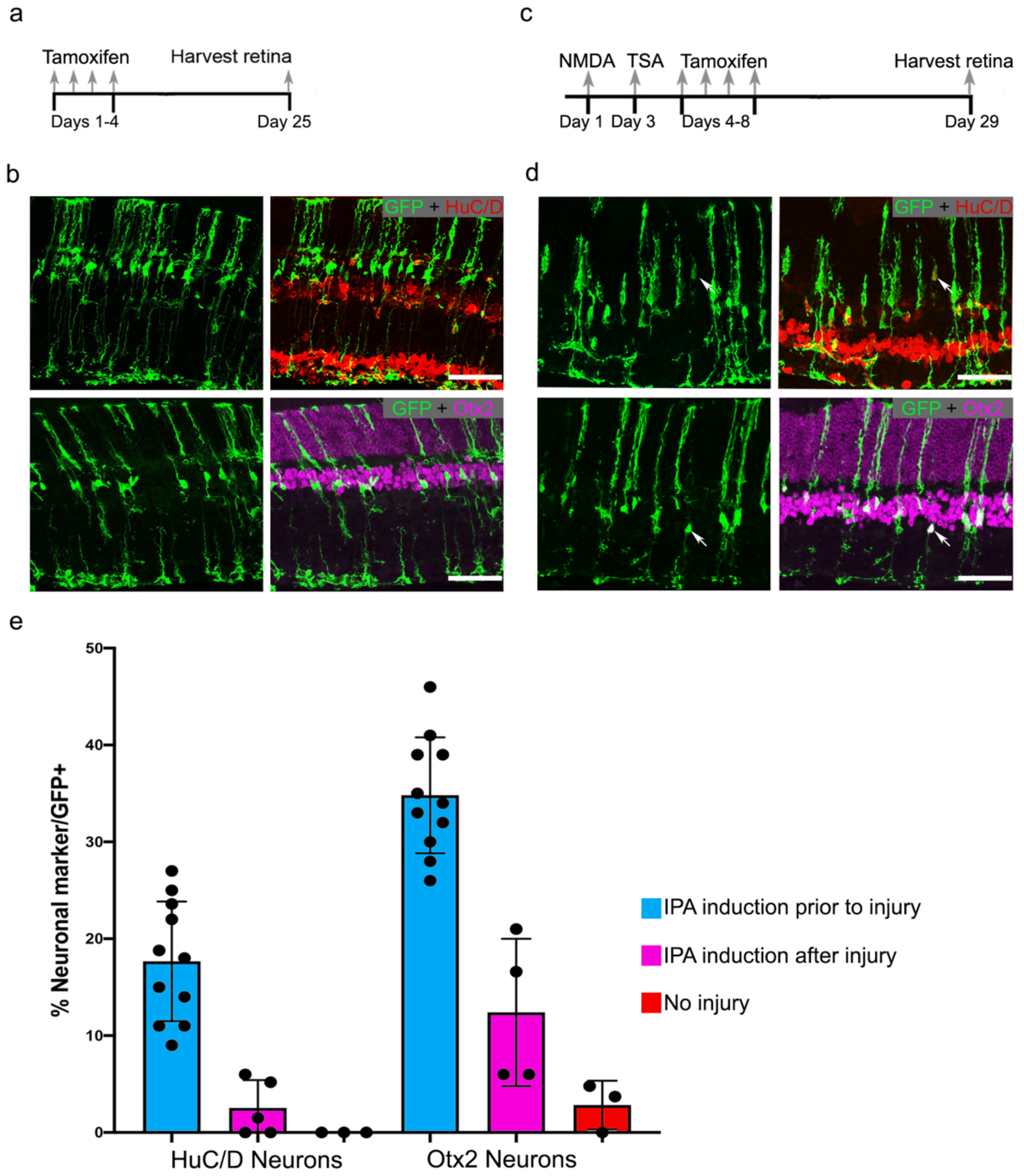

Figure S2: IPA-treatment is most effective at reprogramming MG if induced prior to injury. (a) Experimental paradigm using the transgenic mouse described in Figure1 to test whether *Islet1*/*Pou4f2*/*Ascl1* is able to reprogram MG in the absence of retinal injury. (b) Representative pictures of GFP+ MG showing no induction of the ganglion/amacrine marker HuC/D (red) or the bipolar marker Otx2 (purple). (c) Experimental paradigm to test whether induction of the IPA-factors after NMDA damage and TSA application can induce MG-neurogenesis. (d) Representative pictures of GFP+ MG showing some co-labeling of the neuronal markers HuC/D (red) and Otx2 (purple). (e) Quantification of the percent of MG-derived cells that express HuC/D or Otx2 after IPA-induction prior to injury, after injury, or without injury. Scale bars are 50μm.

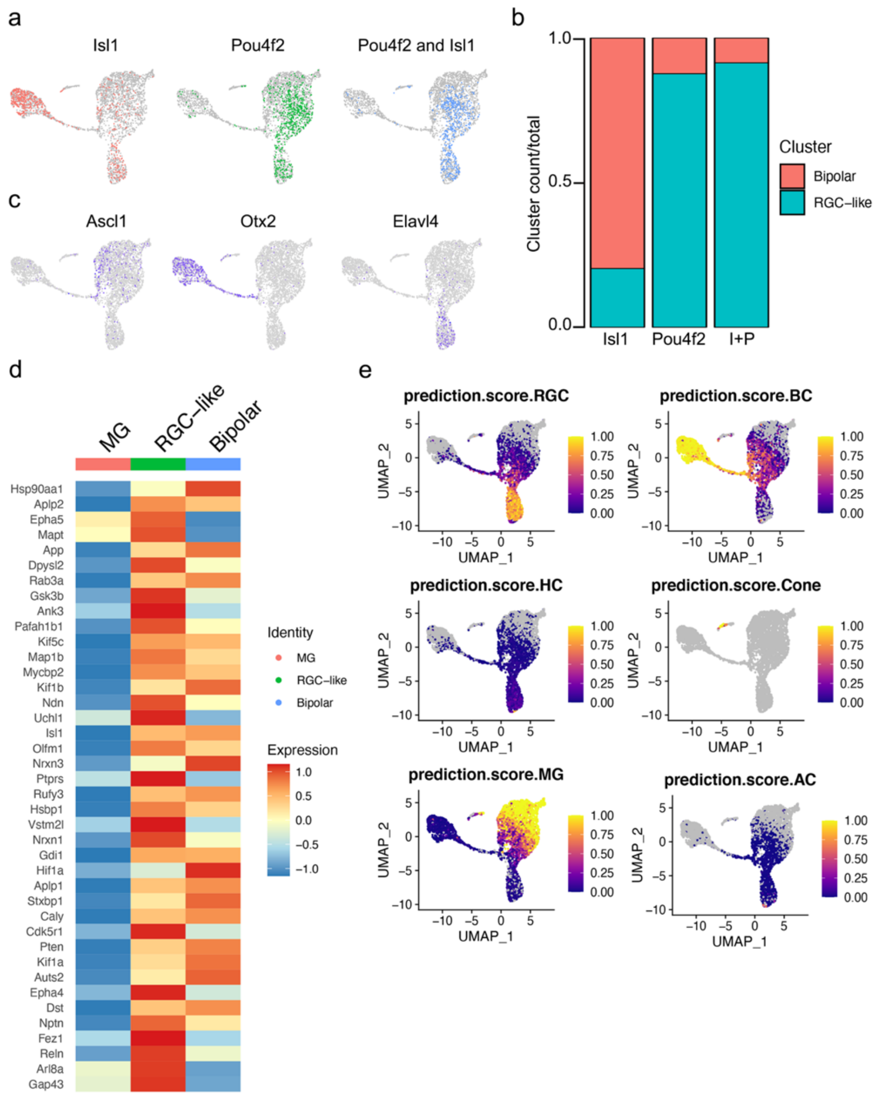

Figure S3: scRNA-seq analysis showing Pou4f2 biases MG-production towards RGC-like neurons. (a) UMAPs of integrated IPA and *Ascl1*-only reprogrammed MG highlighting cells expressing either *Isl1*, *Pou4f2*, or the combination of both. (b) Stacked bar graph quantifying the percent of *Islet1*-only, *Pou4f2*-only, or Islet1/Pou4f2 double-positive cells that end up as MG-derived bipolars or RGC-like neurons. (c) Feature plots highlighting *Ascl1*-expressing cells which get downregulated as they differentiate into bipolar neurons (*Otx2*+) or RGC-like neurons (*Elavl4*+). (d) Heatmap of the top 40 differentially expressed genes between MG and MG-derived neurons found in the GO-terms “axon guidance”, “axonogenesis”, and “axon outgrowth”. (e) Feature plots of prediction scores from Seurat’s label transfer using a reference dataset of randomly sampled cells of each major retinal neuron class and MG, subsetted from mouse retinal development scRNA-seq data[23].

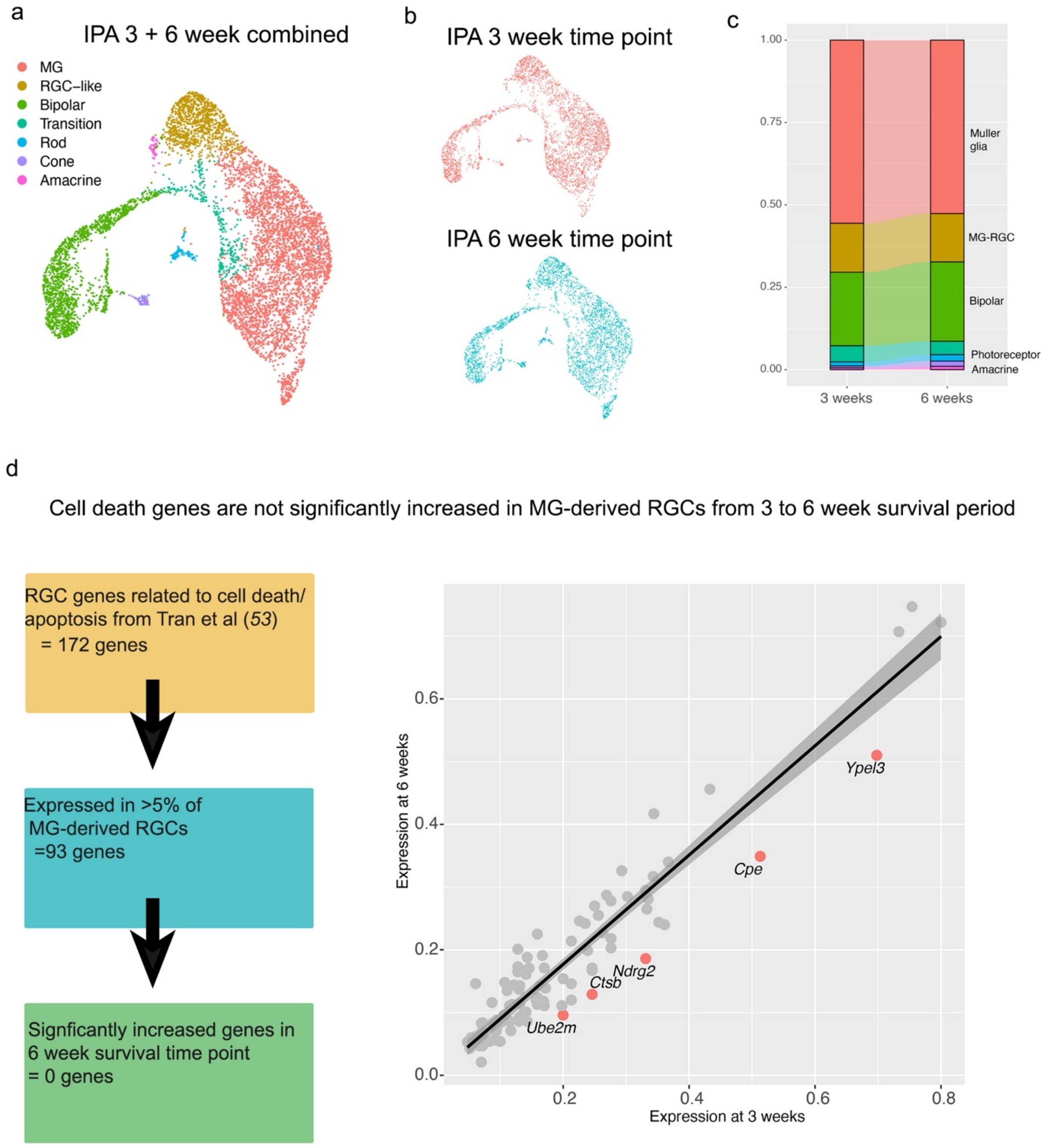

Figure S4: MG-derived RGCs are a stable population over time (a) Combined UMAP of IPA-treated MG from a three and six week end point. (b) Split UMAP showing the distribution of cells in the UMAP in (a) from each time point. (c) Stacked bar graph showing the percentages of each cluster of MG and MG-derived neurons from the three week and six week time point. (d) Analysis of cell death genes found in MG-derived RGC-like neurons.

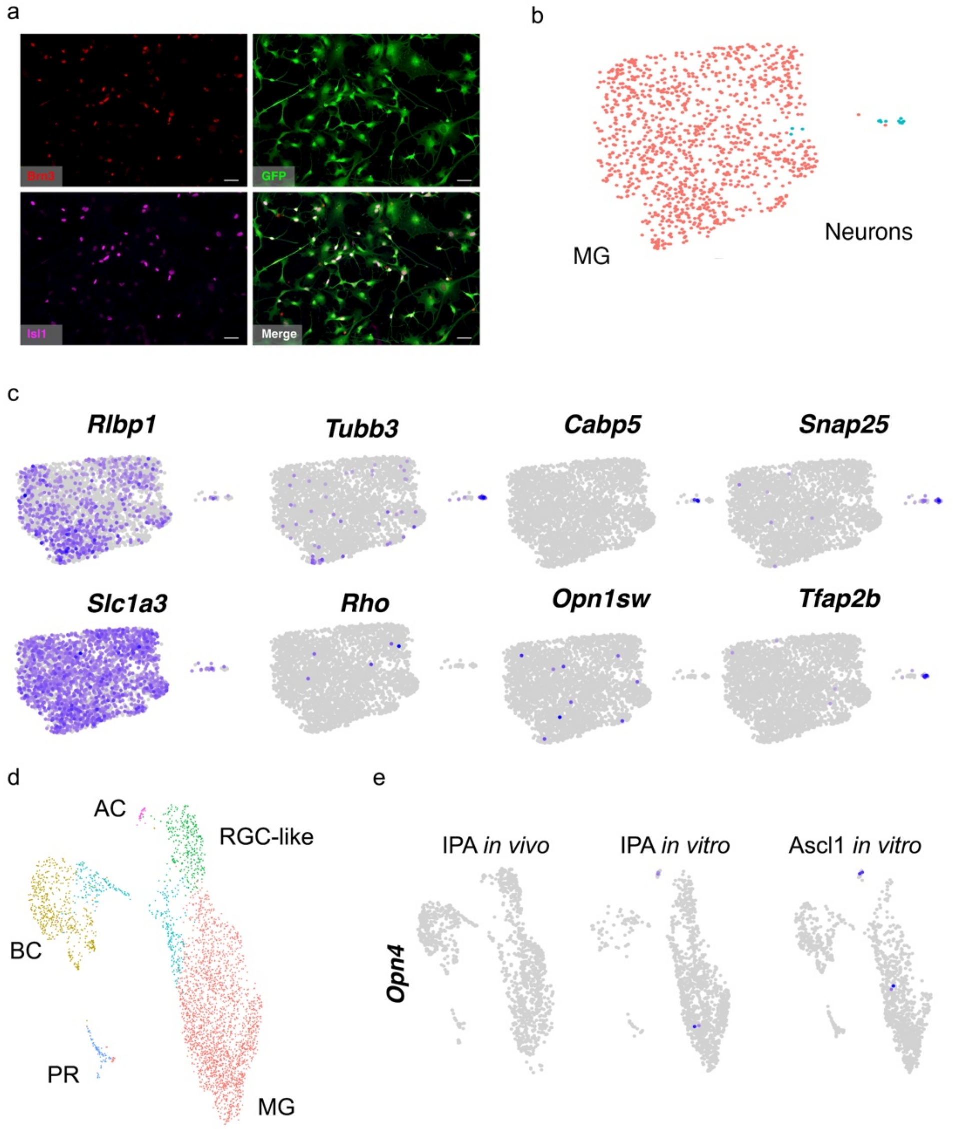

Figure S5: MG from IPA mice express reprogramming factors. (a) Immunofluorescence of IPA MG treated with doxycycline for 5 days showing Ascl1-IRES-GFP, Brn3, and Isl1 co-labeling in most cells (b) UMAP of untreated cultured MG with glial and neuronal cell populations labeled. (c) Markers of MG and various retinal neurons reveal a small population of bipolar and amacrine cells survived dissociation. (d) UMAP of integrated datasets from IPA overexpression *in vivo* and *in vitro,* with Ascl1overexpression *in vitro*. (e) Expression of melanopsin (*Opn4*) in three datasets featured in (d). Scale bars are 50µm. Abbreviations: MG, Müller glia; AC, amacrine cells; BC, bipolar cells; PR, photoreceptors; RGC, retinal ganglion cells

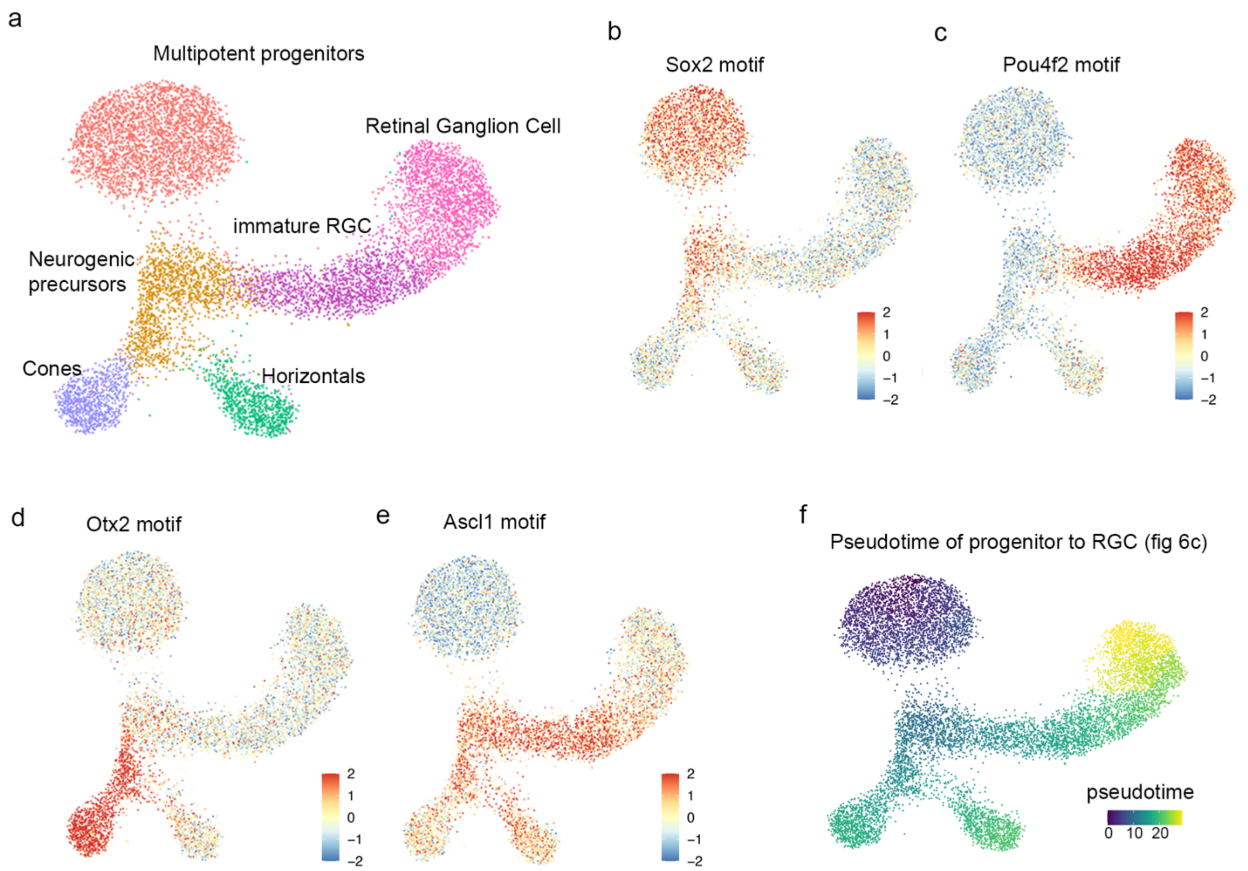

Figure S6: scATAC-seq of the E14 embryonic mouse retina. **a.** UMAP plot of scATAC-seq from E14 embryonic mouse retina. Chromvar scores show the motif accessibility used to identify the clusters of progenitors (**b.** *Sox2*), retinal ganglion cells (**c.** *Pou4f2*), cones (**d.** *Otx2*), and neurogenic precursors (**e.** *Ascl1*). **f.** Pseudotime subset of the transition of retinal progenitor cells to retinal ganglion cells that is further analyzed in **Fig 6**.

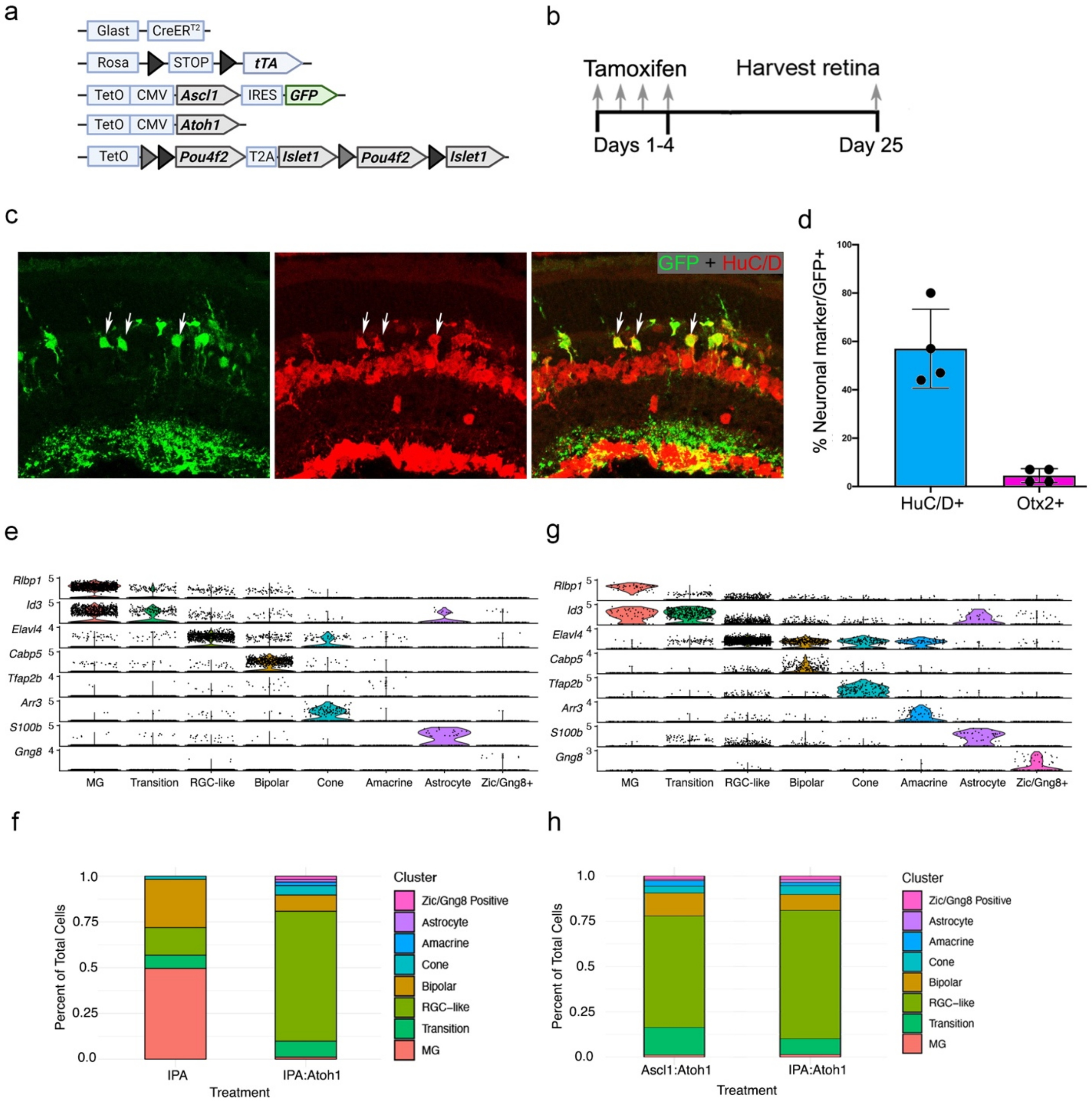

Figure S7: The addition of *Atoh1* to IPA significantly induces MG-derived RGC-like cells and does not require retinal damage. (a) Transgenic mouse construct used for induction of *Ascl1*, *Atoh1*, *Pou4f2*, and *Islet1*. (b) Paradigm to assay whether induction of all four transcription factors can induce MG-neurogenesis in the absence of retinal injury. (c) Representative section of a retina after IPAA treatment showing MG-derived (GFP+) cells express the neuronal marker HuC/D (red). (d) Quantification of the percent of MG-derived cells that express the RGC-like marker HuC/D or the bipolar marker *Otx2* after IPAA treatment without injury. (e-h) Violin plots of markers used to define clusters, and bar plots showing cluster composition of IPA-*IPA*:*Atoh1* (e-f) and *Ascl1*:*Atoh1*-IPA:*Atoh1* (g-h) integrated scRNA-seq data.

REAGENT or RESOURCE	SOURCE	IDENTIFIER	CONCENTRATION
Antibodies			
Goat anti-Brn3	Santa Cruz	Cat#: SC-6026<br>RRID: AB_673441	1:300
Rabbit anti-Calbindin	Millipore	Cat#:AB1778<br>RRID:AB_2068336	1:1000
Rabbit anti-Calretinin	SW Ant	Cat#:7699<br>RRID: AB_10000321	1:500
Chicken anti-GFP	Abcam	Cat#: Ab13970<br>RRID: AB_300798	1:1000
Mouse anti-HUC/D	Invitrogen	Cat#: A-21271<br>RRID: AB_221448	1:500
Mouse anti-Islet1	DSHB	Cat#: 40.2D6<br>RRID: AB_528315	1:50
Mouse anti-Neu/N	Millipore	Cat#: MAB377<br>RRID: AB_2298767	1:1000
Rabbit anti-Neurofilament	Chemicon	Cat#: AB1982<br>RRID:AB_2313731	1:500
Goat anti-OTX2	R&D Systems	Cat#: BAF1979<br>RRID: AB_2157171	1:250
Rabbit anti-SATB1	Abcam	Cat#: Ab109122<br>RRID:AB_10862207	1:300
Goat anti-Sox2	Santa Cruz	Cat#: SC-17320<br>RRID:AB_2286684	1:1000
Rabbit anti-Tuj1	Covance	Cat#: MRB-435p<br>RRID: AB_2313773	1:500
Secondaries			
Donkey anti-chicken 488	Jackson Immuno	Cat#:703-545-155	1:1000
Donkey anti-goat 568	Life Technologies	Cat#: A11057	1:1000
Donkey anti-goat 647	Jackson Immuno	Cat#: 705-605-147	1:1000
Donkey anti-mouse 568	Life Technologies	Cat#: A10037	1:1000
Donkey anti-mouse 647	Jackson Immuno	Cat#: 715-605-150	1:1000
Donkey anti-rabbit 568	Life Technologies	Cat#: A100042	1:1000
Donkey anti-rabbit 647	Thermo Fisher Scientific	Cat#: A-31573	1:1000

Table S1: List of antibodies used in the study. Columns identify the antibody, source, catalog number, RRID identifier and concentration used for immunohistochemistry.

Table S2: Genes associated with peaks enriched in E14 RGCs vs. MG-derived RGC-like cells
